# CDKN2A-mediated molecular subtypes characterize the hallmarks of tumor microenvironment and guide precision medicine in triple-negative breast cancer

**DOI:** 10.3389/fimmu.2022.970950

**Published:** 2022-08-16

**Authors:** Tianyi Cheng, Yingyi Wu, Zhiyu Liu, Yi Yu, Shixue Sun, Min Guo, Baoqing Sun, Chen Huang

**Affiliations:** ^1^ Faculty of Chinese Medicine, State Key Laboratory of Quality Research in Chinese Medicines, Macau University of Science and Technology, Macao, Macao SAR, China; ^2^ Dr. Neher’s Biophysics Laboratory for Innovative Drug Discovery, Macau University of Science and Technology, Macao, Macao SAR, China; ^3^ Guangzhou Municipal and Guangdong Provincial Key Laboratory of Molecular Target and Clinical Pharmacology, The NMPA and State Key Laboratory of Respiratory Disease, School of Pharmaceutical Sciences and the Fifth Affiliated Hospital, Guangzhou Medical University, Guangzhou, China; ^4^ Guangdong Key Laboratory of Animal Conservation and Resource Utilization, Guangdong Public Laboratory of Wild Animal Conservation and Utilization, Institute of Zoology, Guangdong Academy of Sciences, Guangzhou, China; ^5^ Department of Allergy and Clinical Immunology, Department of Laboratory, National Center for Respiratory Medicine, National Clinical Research Center for Respiratory Disease, State Key Laboratory of Respiratory Disease, Guangzhou Institute of Respiratory Health, The First Affiliated Hospital of Guangzhou Medical University, Guangzhou, China

**Keywords:** cuproptosis, immunotherapy, tumor microenvironment, triple-negative breast cancer, cyclin-dependent kinase inhibitor 2A

## Abstract

Currently, breast cancer (BRCA) has become the most common cancer in the world, whose pathological mechanism is complex. Among its subtypes, triple-negative breast cancer (TNBC) has the worst prognosis. With the increasing number of diagnosed TNBC patients, the urgent need of novel biomarkers is also rising. Cyclin-dependent kinase inhibitor 2A (CDKN2A) has recently emerged as a key regulator associated with ferroptosis and cuproptosis (FAC) and has exhibited a significant effect on BRCA, but its detailed mechanism remains elusive. Herein, we conducted the first converge comprehensive landscape analysis of FAC-related gene CDKN2A in BRCA and disclosed its prognostic value in BRCA. Then, an unsupervised cluster analysis based on CDKN2A-correlated genes unveiled three subtypes, namely cold-immune subtype, IFN-γ activated subtype and FTL-dominant subtype. Subsequent analyses depicting hallmarks of tumor microenvironment (TME) among three subtypes suggested strong association between TNBC and CDKN2A. Given the fact that the most clinically heterogeneous TNBC always displayed the most severe outcomes and lacked relevant drug targets, we further explored the potential of immunotherapy for TNBC by interfering CDKN2A and constructed the CDKN2A-derived prognostic model for TNBC patients by Lasso-Cox. The 21-gene–based prognostic model showed high accuracy and was verified in external independent validation cohort. Moreover, we proposed three drugs for TNBC patients based on our model *via* targeting epidermal growth factor receptor. In summary, our study indicated the potential of CDKN2A as a pioneering prognostic predictor for TNBC and provided a rationale of immunotherapy for TNBC, and offered fresh perspectives and orientations for cancer treatment *via* inducing ferroptosis and cuproptosis to develop novel anti-cancer treatment strategies.

## Introduction

Nowadays, breast cancer (BRCA) is the worldwide leading cause of cancer incidences and the second leading cause of cancer-related death (1). Triple-negative breast cancer (TNBC), which is distinguished by the absent expression of human epidermal growth factor receptor 2 (Her2) and estrogen receptor/progesterone receptor (ER/PR), is the most invasive subtype with the highest mortality rate accounting for approximately 15% of all BRCA (2). The mortality rate is up to 40% within 5 years after the first diagnosis and distant metastasis will occur in approximately 46% of TNBC patients (3). Hence, the incidence and mortality of TNBC make it necessary to explore reliable predictive biomarkers, construct more promising prognostic models and develop novel drugs that target at the known molecular pathways.

Cyclin-dependent kinase inhibitor 2A (CDKN2A), a cyclin-dependent kinase inhibitor gene, that encodes the p16 protein involved in the regulation of cell cycle pathways, is known as a tumor suppressor (4). CDKN2A can inactivate the retinoblastoma protein by binding to and inactivating the cyclin D-cyclin-dependent kinase 4 complex (5). The expression of this gene is verified to cause cell cycle arrested in the G1 phase, inhibit cell proliferation, promote tumor cell apoptosis, and increase tumor cell chemotherapy sensitivity (6). Recent studies have pointed out that CDKN2A is correlated with ferroptosis (7) and cuproptosis (8) (FAC), which are both novel types of regulated cell death that their occurrence was ion-dependent. Ferroptosis indicates an oxidative cell death resulting from the deterioration of antioxidant function and accretion of lipid reactive oxygen species (ROS) (9). Recent attention has been brought to a brand-new cell death mode identified as cuproptosis, which indicates that the excess copper can trigger proteotoxic stress and death in cells through the combination with lipoylated components of the tricarboxylic acid (TCA) cycle (8). These ion-dependent cell death different from apoptosis, necrosis, and autophagy can contribute to a burgeoning field that promising cancer drugs are designed based on the induction of ferroptosis (10) and cuproptosis (8).

Additionally, researchers have discovered that the malfunctioning of CDKN2A in BRCA has promoted the discovery of many CDK inhibitors (11). The role of CDKN2A in BRCA cannot be ignored and needs further investigations. However, the investigations about the role of CDKN2A in BRCA are limited, we are thereby unable to comprehensively elaborate the biological function of CDKN2A. Hence, we conducted the first converge comprehensive landscape analysis of FAC-related gene CDKN2A in BRCA, including expression, prognostic values, DNA methylation, tumor microenvironment (TME) analysis, and drug sensitivity of CDKN2A in BRCA. Immediately afterward, unsupervised cluster analysis revealed the difference in immunological analysis and FAC status of CDKN2A-associated genes among 3 groups, namely cold-immune subtype, IFN-γ activated subtype, and FTL-dominant subtype, groundbreakingly laying a foundation for the application of immunotherapy and FAC regulators in BRCA. Given the strong association between TNBC and CDKN2A, as well as the fact that the most clinically heterogeneous TNBC always displayed the most severe outcomes and lacked relevant drug targets, we further explored the potential of immunotherapy for TNBC by regulating CDKN2A and constructed the CDKN2A-derived prognostic model for TNBC patients by machine learning, aiming to predict the prognosis of TNBC patients and provide the guidance on their long-term disease outlook and design of treatment strategies.

## Materials and methods

### Data collection

BRCA data was downloaded from the UCSC Xena data mining platform (http://xena.ucsc.edu/), which included the messenger RNA (mRNA) expression matrix from The Cancer Genome Atlas (TCGA), as well as the clinical information of 33 cancer types. Gene expression profile of BRCA patients and their corresponding clinical information were obtained. Specimens without survival information were excluded during this study and FPKM values of RNA-Seq were log2 transformed. In total, 1072 BRCA patients and included 185 TNBC samples were retained for subsequent analysis.

We extracted and exhibited detailed clinical information of 1072 BRCA patients as shown in **Table 1**: age, sex, pathological stage, estrogen receptor (ER) status, progesterone receptor (PR) status, human epidermal growth factor 2 (HER2) status, T/N/M stage, and adjuvant chemotherapies. Additionally, gene expression array GSE58812 containing 107 TNBC patients and their corresponding clinical data was retrieved from the Gene Expression Omnibus (GEO) (https://www.ncbi.nlm.nih.gov/geo/) to externally validate the CDKN2A-derived prognostic model constructed in our study. Gene expression array GSE173839 containing clinical information of 100 BRCA patients was used to evaluate the status of immunotherapy response between high and low CDKN2A expression groups in TNBC patients.

**Table 1:** Clinical pathological characteristics of extracted BRCA patients.

Characteristic	Group	No. of cases (%)
Age (years)	<60	570 (53.17%)
	≥60	500 (46.64%)
	Unknown	2 (0.18%)
Sex	Female	1059 (98.78%)
	Male	12 (1.12%)
	Unknown	1 (0.09%)
Pathological Stage	Stage I	176 (16.42%)
	Stage II	607 (56.62%)
	Stage III	245 (22.85%)
	Stage IV	20 (1.86%)
	Stage X	12 (1.12%)
	Unknown	12 (1.12%)
Pathological T	T1	274 (25.56%)
	T2	621 (57.93%)
	T3	133 (12.40%)
	T4	40 (3.73%)
	TX	3 (0.27%)
	Unknown	1 (0.09%)
Pathological N	N0	502 (46.83%)
	N1	355 (33.11%)
	N2	118 (11.01%)
	N3	76 (7.09%)
	NX	20 (1.86%)
	Unknown	1 (0.09%)
Metastasis	M0	896 (83.58%)
	M1	22 (2.05%)
	MX	153 (14.27%)
	Unknown	1 (0.09%)
ER	Positive	789 (73.60%)
	Negative	232 (21.64%)
	Unknown	51 (4.75%)
PR	Positive	684 (63.80%)
	Negative	334 (31.15%)
	Unknown	54 (5.04%)
HER2	Positive	162 (15.11%)
	Negative	547 (51.02%)
	Unknown	363 (33.86%)
Adjuvant therapy	No	1056 (98.51%)
	Yes	13 (1.21%)
	Unknown	3 (0.27%)
OS Status	Living	921 (85.91%)
	Dead	150 (13.99%)
	Unknown	1 (0.09%)

### Landscape analysis of CDKN2A in BRCA

To comprehensively investigate the biological role of CDKN2A in BRCA, we started with the pan-cancer analysis of the CDKN2A expressions *via* Tumor Immune Estimation Resource (TIMER) (https://cistrome.shinyapps.io/timer/) database (12). Then, the expression levels of CDKN2A in BRCA, as well as normal tissues were validated in Gene expression profiling interactive analysis (GEPIA) (http://gepia.cancer-pku.cn/) sequencing expression (13). Subsequently, Human Protein Atlas (HPA) (https://www.proteinatlas.org/) provided the immunohistochemical images of CDKN2A expression in BRCA samples (14). To further explore the biological role of CDKN2A in BRCA, UALCAN (http://ualcan.path.uab.edu/index.html) (15) and MEXPRESS (http://mexpress.be) (16) were utilized to analyze the DNA promoter methylation status of CDKN2A in BRCA and the effects of methylation of CDKN2A on clinical stages in BRCA. Besides, cBio Cancer Genomics Portal (cBioPortal) (http://cbioportal.org/) and Catalogue of Somatic Mutations In Cancer (COSMIC) (https://cancer.sanger.ac.uk) were conducted to analyze the mutation status of CDKN2A in BRCA (17, 18). TIMER and Tumor and Immune System Interaction Database (TISIDB) (http://cis.hku.hk/TISIDB) database (19) were used to comprehensively explore the relationship between CDKN2A expression and immune infiltration. Moreover, CellMiner (20) was performed to evaluate the relationship between CDKN2A and drug sensitivity, looking for the targeted therapies for BRCA patients.

### Unsupervised clustering of CDKN2A-associated differentially expressed genes

Based on the integration of CDKN2A strongly associated genes and differential genes, we used the R package “ConsensusClusterPlus” (https://bioconductor.org/packages/ConsensusClusterPlus/), to perform an unsupervised cluster analysis. After 1,000 iterations of the consensus clustering algorithm, the number of optimal clusters was confirmed according to the Item-Consensus plot, cumulative distribution function curves, and the k-means clustering algorithm. Three unsupervised clusters (cold-immune cluster, IFN-γ activated cluster, and FTL-dominant cluster) were selected for subsequent analysis.

### Profiling analysis of tumor microenvironment

As a generic computational method of calculating cell fractions from gene expression data, Cell-type Identification By Estimating Relative Subsets Of RNA Transcripts (CIBERSORT) (21), was separately used to analyze the proportions of 22 infiltrating immune cells in high and low CDKN2A expression groups in BRCA, as well as between 3 groups from the unsupervised cluster. The ESTIMATE approach calculated the stromal, immune, and ESTIMATE scores of three groups, predicting the level of stromal cells and infiltrating immune, which constructed the basis of tumor purity (22). The immunotherapy-related pathways, immune checkpoints, and 122 immunomodulators, including chemokines, receptors, MHCs, and immune stimulators were obtained from previous studies (23–25). Single-Sample Gene Set Enrichment Analysis (ssGSEA) was performed to derive the enrichment score of all steps *via* the R package “GSVA” (26).

### Evaluation of immunotherapy response sensitivity in TNBC patients with different CDKN2A expression

Immunophenoscore (IPS) is a generic machine learning-based algorithm for quantifying tumor immunogenicity, which is measured grounded in the gene expression of representative cell types, including immunomodulators, immunosuppressive cells, MHC molecules, and effector cells (27). The IPS from The Cancer Immunome Atlas (TCIA) (https://tcia.at/) was calculated in the high and low CDKN2A expression groups in TNBC patients. In general, the higher IPS indicates a better immunotherapy response. Subsequently, to further predict the sensitivity to immunotherapy response, GSE173839 was used to evaluate the status of immunotherapy response between high and low CDKN2A expression subpopulations in TNBC patients.

### Exploration of functional annotation of CDKN2A-associated genes

Gene Ontology (GO) and Kyoto Encyclopedia of Genes, Genomes (KEGG) functional enrichment analysis confirmed the functions of CDKN2A-associated genes *via* the R language “cluster Profiler” package (https://guangchuangyu.github.io/software/clusterProfiler/). Additionally, gene sets “h.all.v7.5.1.symbols.gmt” were obtained from Molecular Signatures Database (MSigDB) (https://software.broadinstitute.org/gsea/downloads.jsp) and were used for calculations of 50 hallmark tumor-related pathways. Moreover, oxidative stress caused by the accumulation of lethal ROS is the recognized process of ferroptosis. But, due to the definition of cuproptosis being relatively avant-garde, the controversy of whether cuproptosis is a form of cell death independent of other cell death modes or not still exists. Therefore, based on the GO enrichment analysis and previous studies, we collected 7 pathways implicated in ferroptosis and cuproptosis *via* literature retrieval and vicariously evaluated their activities in BRCA by ssGSEA analysis, including fatty acids degradation (28), inflammatory response (29), oxidative stress (9), positive regulation of MAPK cascade (30), regulation of mitochondrial membrane potential (9), TCA cycle (8) and VEGF signaling pathway (31). Moreover, Metascape (32) was used to analyze the functional annotation of 413 survival-related differentially expressed genes (SDEGs), aiming to reveal the biological mechanism of the influence of CDKN2A on the survival status of TNBC patients. Besides, Search Tool for the Retrieval of Interacting Genes (STRING) (https://string-db.org/) database (33) was utilized to gather and construct data about protein-protein interaction (PPI) of CDKN2A and genes constructing the model.

### Weighted gene co-expression network analysis based on RNA-seq data

After deleting the outliers in the gene expression matrix, the TCGA-A7-A0DC-01A sample and the TCGA-A2-A3XV-01A sample were expelled. Based on a scale-free topology with R2 = 0.85, the adjacency matrix was defined by using soft thresholding with power β =6, to identify and build the different co-expression gene modules in BRCA samples. Then, the CDKN2A-derived genes were clustered based on a topological overlap matrix (TOM)-based dissimilarity measure, and the cluster dendrogram of all these genes was constructed by R package “WGCNA” (34). Every identified co-expression module was labeled with a different color. Then, we conducted principal component analysis (PCA) of each module, extracted and summarized the gene co-expression based on the eigengene external traits that included TNBC and the status of ferroptosis and cuproptosis (substituted by the scores of oxidative stress, regulation of mitochondrial membrane potential and TCA cycle). We further calculated the correlation between each eigengene external trait and each module with the biweight midcorrelation (bicor) that could offer robust correlations with minor weight given to outlier measures (35, 36). Subsequently, we selected genes in modules that possessed the strongest relationship with TNBC and FAC as the input for the Least Absolute Shrinkage and Selection Operator (LASSO) regression analysis.

### Construction and validation of the CDKN2A-derived prediction model

Based on the genes from WGCNA, we sequentially developed univariate Cox, LASSO regression *via* the R package “glmnet” to construct the CDKN2A-derived prognostic model (37). The risk score was calculated *via* the following formula:


$$Risk\;Score\;=\sum_{i=1}^{n}Coef_{i}\times exp_{i}$$

The Coef_i_ represented the risk coefficients of each gene weighted by LASSO-Cox model, and exp_i_ indicated the expression of each gene in our study. Then, the Kaplan–Meier survival analysis was developed to evaluate the difference in survival between low and high risk-score groups through R package “survival”. Subsequently, we used the time-dependent receiver operating characteristic (ROC) curve to appraise the performance of the CDKN2A-derived model. Further, to test whether risk score could be an independent prognostic predictor of TNBC patients, univariate Cox and multivariate Cox regression analyses were conducted with risk score, sex, age, metastasis status, tumor stage and pathological status as variables. Ultimately, external validation of the CDKN2A-derived prognostic model was performed *via* the clinical data of 107 TNBC patients contained in GSE58812.

### Potential drug prediction based on the CDKN2A-derived model

Drug sensitivity data of diverse cell lines and corresponding gene-expression data from three databases were used to perform the drug sensitivity analysis based on the CDKN2A-derived signature, including GDSC (Genomics of Drug Sensitivity in Cancer), PRISM (Profiling Relative Inhibition Simultaneously in Mixtures) and CTRP (Cancer Therapeutics Response Portal) (38) (39). AUC values functioned as a measure of drug sensitivity and drugs with missing AUC values more than 80% were excluded. Based on the different drug reactions of high and low groups, drugs with Padj value less than 0.05 were screened out. The compound overlapping in the outcomes of PRISM, CTRP, and GDSC analyses may serve as a potential treatment for the certain subpopulation.

### Statistical analysis

Correlations were analyzed *via* Pearson correlation except for the part of WGCNA using bicor. Statistical analyses were conducted using Kruskal–Wallis, Wilcoxon, chi-square test, and Tuckey’s honestly significant difference and differences were considered significant at P value < 0.05.

## Results

### Landscape analysis of CDKN2A hints at prognostic value and its association with drug sensitivity in BRCA

**Figure 1:**
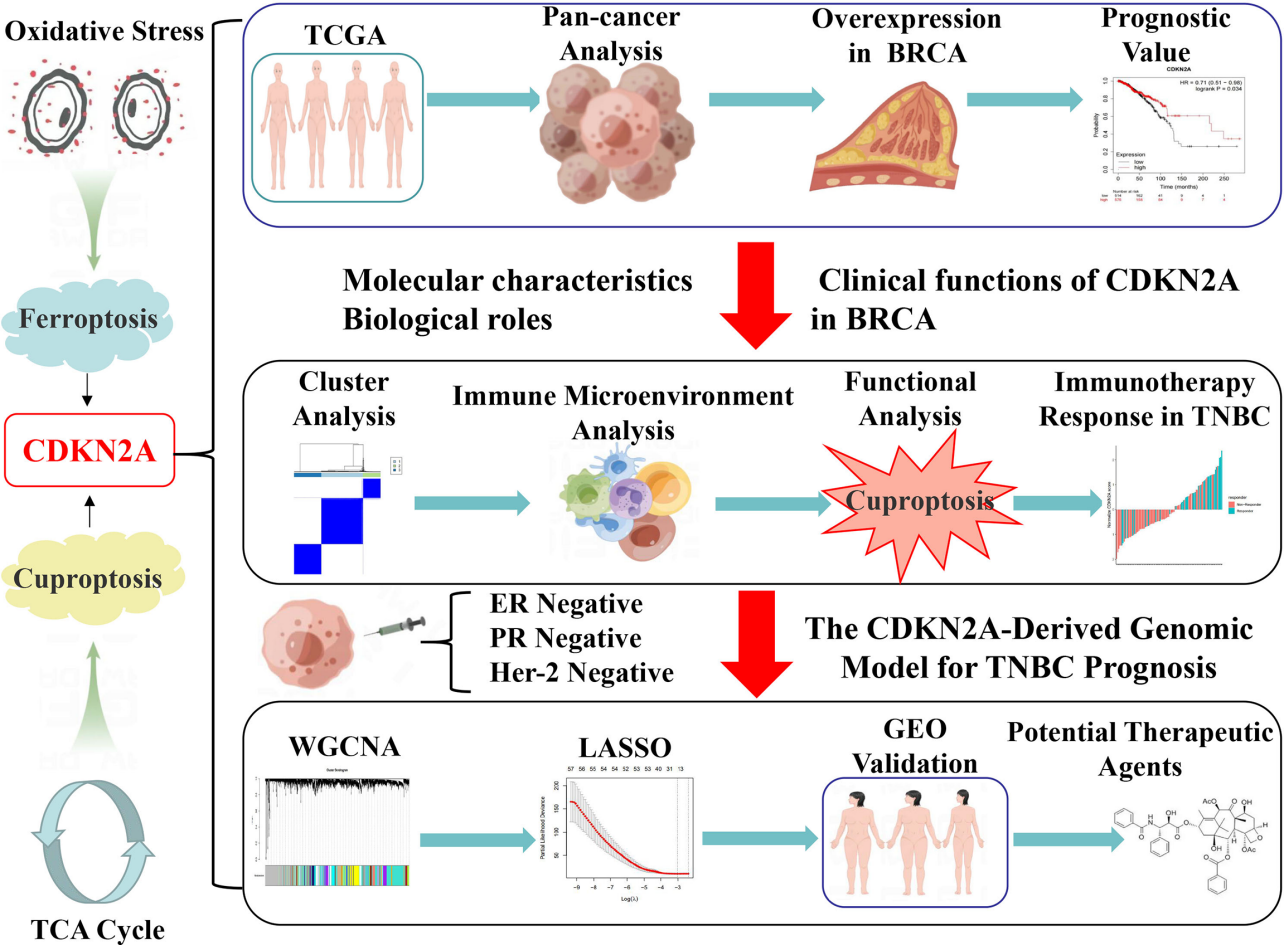
The flow chart of our study.

**Figure 2:**
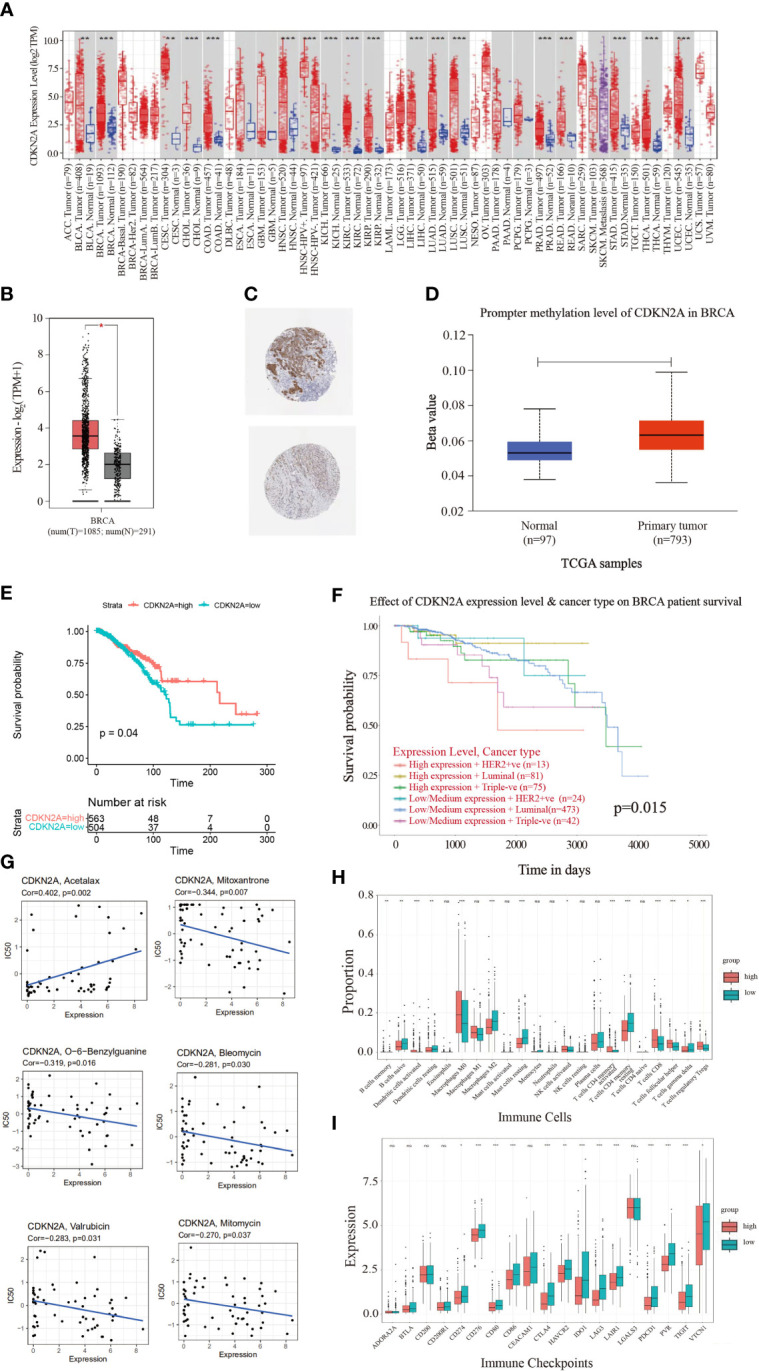
The landscape analysis of overexpressed CDKN2A in BRCA. **(A)** The difference in expression of CDKN2A between various malignant cancer types from the cancer genome map (TCGA) database across TIMER database. CDKN2A was upregulated in bladder urothelial Carcinoma (BLCA), breast invasive carcinoma (BRCA), cervical squamous cell carcinoma and endocervical adenocarcinoma (CESC), cholangiocarcinoma (CHOL), colon adenocarcinoma (COAD), head and neck squamous cell carcinoma (HNSC), kidney chromophobe (KICH), kidney renal clear cell carcinoma (KIRC), kidney renal papillary cell carcinoma (KIRP), liver hepatocellular carcinoma (LIHC), lung adenocarcinoma (LUAD), lung squamous cell carcinoma (LUSC), Prostate adenocarcinoma (PRAD), Rectum adenocarcinoma (PEAD), Stomach adenocarcinoma (STAD), Thyroid carcinoma (THCA) and Uterine Corpus Endometrial Carcinoma (UCEC). (*P < 0.05. **P < 0.01. ***P < 0.001). **(B)** CDKN2A was significantly upregulated in BRCA by GEPIA database. **(C)** Representative immunohistochemical images of CDKN2A in BRCA tissues. **(D)** Promoter methylation levels of CDKN2A in normal tissues and primary BRCA tissues in the UALCAN database. **(E)** The Kaplan-Meier curves of OS for low and high expression of BRCA patients. **(F)** The prognostic values of CDKN2A in different BRCA subtypes. **(G)** Scatter plots depict the relationship between CDKN2A expression and drug sensitivity in BRCA. **(H)** Comparison of infiltration of immune cells between high and low CDKN2A expression groups in BRCA. **(I)** Comparison of immune checkpoints expression between high and low CDKN2A expression groups in BRCA. The P values of the figure are shown as follows: *P < 0.05. **P < 0.01. ***P < 0.001. ns (not significiant, P > 0.05).

**Figure 1** illustrates the flow chart of the present study. In the comparisons of multiple cancers with corresponding normal tissues, CDKN2A exhibited a significant overexpression in bladder urothelial carcinoma (BLCA), BRCA, cervical squamous cell carcinoma and endocervical adenocarcinoma (CESC), cholangiocarcinoma (CHOL), colon adenocarcinoma (COAD), head and neck cancer (HNSC), kidney chromophobe (KICH), kidney renal clear cell carcinoma (KIRC), kidney renal papillary cell carcinoma (KIRP), liver hepatocellular carcinoma (LIHC), lung adenocarcinoma (LUAD), lung squamous cell carcinoma (LUSC), prostate adenocarcinoma (PRAD), rectum adenocarcinoma (READ), stomach adenocarcinoma (STAD), thyroid carcinoma (THCA), and uterine corpus endometrial carcinoma (UCEC) (**Figure 2A**). GEPIA database was used to further confirm that CDKN2A was notably upregulated in BRCA (**Figure 2B**). Immunohistochemistry outcomes from the HPA database illustrated that the protein level of CDKN2A was significantly increased in BRCA tissue (**Figure 2C**). As a fundamental constituent element of epigenetics, DNA methylation modification plays a vital role in silencing the expressions of methylated genes. Our data indicated that CDKN2A was notably hypermethylated in BRCA (**Figure 2D**), especially in the luminal subtype and TNBC subtype (**Supplementary Figure S1C**). BRCA patients with CDKN2A hypermethylation possessed a relatively undesirable clinical outcome (P = 0.0527833983) (**Supplementary Figure S1D**), which needs further investigations. Additionally, the hypermethylation of CDKN2A was positively correlated with the tumor progression, verifying that CDKN2A may be a crucial impact factor in the stage and grade of BRCA (**Supplementary Figures S1A, B**). Besides, the COSMIC database was conducted to evaluate the mutation type of CDKN2A. (**Supplementary Figures S2C, D**). Missense substitutions notably occupied the largest portion, accounting for 38.59%. Next, nonsense substitutions occurred in 30.54%. Frameshift deletions occupied 9.81% of the samples and frameshift insertions occupied 6.40% of the samples. Moreover, our results indicated that the substitution mutations chiefly occurred at C > T (44.24%), G > A (20.45%), G > T (14.91%), and C > A (5.5%). Additionally, cBioPortal database revealed that the mutation frequency of CDKN2A in BRCA was 0.5% and the mutation of CDKN2A had no effects on the prognosis of BRCA patients (P > 0.05) (**Supplementary Figures S2A, B**).

To further explore the molecular characteristics of CDKN2A in BRCA, we performed the relationship between CDKN2A and tumor-infiltrating immune cells using the CIBERSORT algorithm. Our results demonstrated that BRCA patients with high expression of CDKN2A exhibited an increased infiltration level of most immune cells in BRCA, including activated dendritic cells, M0 macrophages, activated NK cells, activated memory CD4 + T cells, CD8 + T cells, follicular helper T cells, and regulatory T cells (**Figure 2H**). Moreover, the expression of CDKN2A was positively associated the expression of multiple immune checkpoints, including CTLA4, PDCD1, PVR, TIGIT, and so on in BRCA (**Figure 2I**). Our data from Timer 2.0 and TISIDB database also indicated that CDKN2A expression could significantly affect the immune infiltration status and immune microenvironment of BRCA. The expression of CDKN2A was respectively correlated with the infiltration abundances of macrophages in basal-like BRCA, myeloid dendritic cells and CD8+ T cells in luminal A (**Supplementary Figure S3A**). Besides, CDKN2A expression was also correlated with CCL5, CCL7, CCL8, CXCL16, etc (**Supplementary Figure S3B**).

Survival analysis was further indicative that BRCA patients with high expression of CDKN2A had better overall survival (OS) than those with low CDKN2A expression (**Figure 2E**). The prognostic values of CDKN2A expression in different subtypes of BRCA also presented a significant differentiation (**Figure 2F**). The genetic alterations caused by the heterogeneity of BRCA may also affect the responses to target agents (40). Improved reliable biomarkers for targeted treatment are needed. Consequently, the relationship between CDKN2A expression and drug sensitivity was conducted, exploring the clinical roles of CDKN2A. Our data indicated that the expression of CDKN2A was positively connected with sensitivity to acetalax (**Figure 2G**). Otherwise, CDKN2A presented a negative correlation to sensitivity to mitoxantrone, O-6-Benzylguanine, bleomycin, valrubicin, and mitomycin. In conclusion, CDKN2A has the potential of being a predictive marker of the aforementioned agents.

### Characterizations of CDKN2A-mediated genes reveal linkage of CDKN2A to TME and prognostic value in TNBC

**Figure 3:**
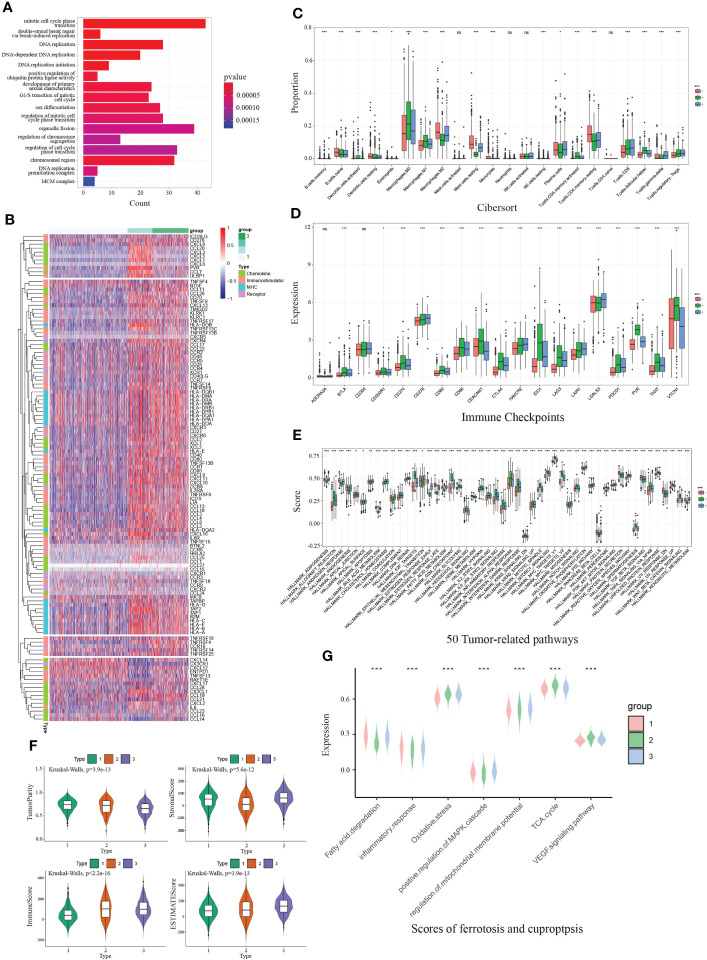
The immunological and functional analysis of CDKN2A among 3 groups from unsupervised clustering in BRCA. **(A)** The GO enrichment analysis revealed the function of CDKN2A-mediated genes. **(B)** The heatmap depicted each BRCA patient with a difference of a corresponding enrichment of 122 immunomodulators. **(C)** Comparison of infiltration of immune cells between 3 groups. **(D)** Comparison of immune checkpoints expression between 3 groups in BRCA. **(E)** Comparison of 50 tumor-related pathways between 3 groups in BRCA. **(F)** Comparison of estimate score, immune score, stromal score, and tumor purity between 3 groups in BRCA. **(G)** Comparison of scores of ferroptosis and cuproptosis between 3 groups in BRCA. The P values of the figure are shown as follows: *P < 0.05. **P < 0.01. ***P < 0.001. ns (not significiant, P > 0.05).

To depict the crosstalk between CDKN2A and TME in BRCA, two strategies were initially proposed to detect CDKN2A-medicated genes. Concretely, 737 CDKN2A-correlated genes were obtained using Pearson correlation analysis (|corrcoef| > 0.4). Then we divided 1072 TCGA-BRCA patients into four quartiles ranked by their expression of CDKN2A and identified 228 differentially expressed genes (DEGs) between the two quartiles groups with the highest and lowest expression (|logFC| > 1, adjust P < 0.05). Combined with 737 CDKN2A-correlated genes, a total of 885 CDKN2A-mediated genes were figured out. Subsequent functional analysis indicated that these genes might involve in mitotic cell cycle phase transition, double-strand break repair *via* break-induced replication, DNA replication, positive regulation of ubiquitin protein ligase activity, etc (**Figure 3A**, **Supplementary Figure S4A**). Afterwards, an unsupervised cluster analysis was conducted based on CDKN2A-medicated genes *via* R package “ConsensusClusterPlus”. As a result, 1072 BRCA patients were divided into 3 subgroups with optimal stability of the classification. Through CIRBERSORT analysis, Subgroup 1 was closely associated with an increased infiltration of resting dendritic cells, M2 Macrophages, resting mast cells, eosinophils, monocytes, and resting memory CD4+T cells (**Figure 3C**), equivalent to the phenotype with immunosuppressive characteristic (41), which, thereby, was defined as cold-immune subtype. Similarly, subgroup 2 was defined as IFN-γ activated subtype due to its elevated infiltration of M0 and M1 Macrophages, activated dendritic cell, CD8 T cells, follicular helper T cells and activated memory CD4+T cells (**Figure 3C**), which correspond to the active-immune phenotype (42, 43). Subtype 3 was characterized by the highest expression of FTL, namely FTL-dominant subtype. Also, three subgroups were found to present the conspicuous discrepancy of expression differences of immune checkpoints genes (**Figure 3D**). In particular, IFN-γ activated subtype exhibited an elevated expression level of multiple immune checkpoints, including CD274, CTLA4, PDCD1, PVR, TIGIT and VTCN1, etc. Moreover, IFN-γ activated subtype correlated the relatively highest scores of certain immunotherapy-related pathways, especially in IFN-γ pathway (**Supplementary Figure S4B**), which was another reason for naming it. Furthermore, the heatmap depicted each BRCA patient with a corresponding enrichment of 122 immunomodulators among three groups, including chemokines, receptors, MHCs and immune stimulators (**Figure 3B**). As demonstrated in the chart, cold-immune subtype could be insinuated as an immunologically “cold” phenotype. Notably, IFN-γ activated subtype relatively possessed the highest immune activity. These findings were consistent with results of ESTIMATE analysis (**Figure 3F**).

Notably, IFN-γ activated cluster was significantly associated with numerous pathways, such as MYC targets, inflammatory response, IL6/JAK/STAT3 signaling pathway and IFN-γ response, which was consistent with our way of naming it (**Figure 3E**). Since CDKN2A is the ferroptosis and cuproptosis-related gene, we collected seven pathways implicated in ferroptosis and cuproptosis *via* literature retrieval and outcomes of GO enrichment analysis, aiming to vicariously evaluate their activities in patients with BRCA by ssGSEA. The results demonstrated that ssGSEA scores of those FAC-related pathways significantly differed among three subgroups (**Figure 3G**). Notably, the FTL-dominant subtype possessed the relatively highest scores of oxidative stress, demonstrating its elevated activity in ferroptosis.

**Figure 4:**
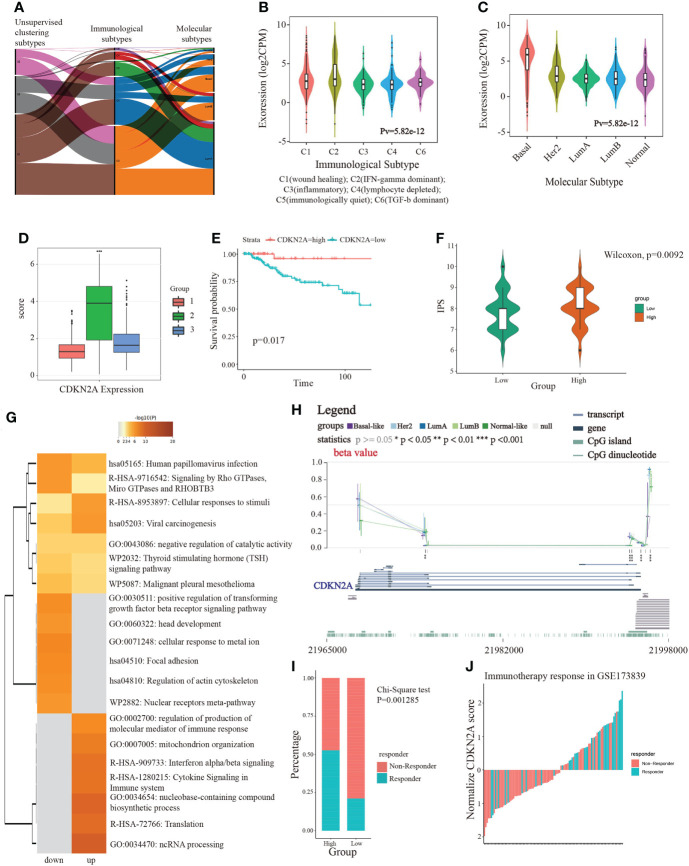
The linkage of CDKN2A to immunotherapy and TNBC. **(A)** The correlation between molecular subtypes, immunological subtypes, and our unsupervised subtypes in BRCA. **(B)** The relationship between CDKN2A expression and immunological subtypes of BRCA. **(C)** The relationship between CDKN2A expression and molecular subtypes of BRCA. **(D)** The comparison between CDKN2A expression and unsupervised subtypes of BRCA. **(E)** The survival value of CDKN2A in TNBC. **(F)** The comparison between IPS score and high and low CDKN2A expression subpopulations in TNBC. **(G)** The function annotation analysis of up-regulated and down-regulated SDEGs in high and low CDKN2A expression subpopulations. **(H)** The comparison between methylation status of CDKN2A and molecular subtypes of BRCA. **(I, J)** The correlation between immunotherapy response status and CDKN2A expression in TNBC *via* chi-square test. The P values of the figure are shown as follows: *P < 0.05. **P < 0.01. ***P < 0.001.

Next, we investigated the correlation between molecular subtyping, immunological subtyping, and our unsupervised subtyping in BRCA. Unexpectedly, we found that the majority of patients (approximately 98.4%) of basal-like subtype were part of IFN-γ activated subtype (**Figure 4A**). More subtly, IFN-γ activated subtype chiefly belonged to the C2 subtype that was dominated by IFN-γ (**Figures 4B, C**). Our results also demonstrated that CDKN2A more significantly overexpressed in TNBC patients than non-TNBC patients (**Supplementary Figure S5A**). Notably, our analysis demonstrated the CDKN2A expression was relatively the highest in our S2 subtype (**Figure 4D**), which suggested the close correlation between CDKN2A and TNBC (basal-like) subtype. This point was supported by subsequent survival analysis, which indicated that TNBC patients with low expression of CDKN2A exhibited an undesirable clinical outcome (**Figure 4E**). Moreover, CDKN2A also exhibited four methylation sites with statistical significance among the molecular subtypes (**Figure 4H**). Subsequently, we aimed at exploring the underlying biological mechanism behind the survival difference between the two groups through difference analysis and function annotation analysis. We further identified 413 survival-related differentially expressed genes (SDEGs) between two groups with high and low CDKN2A expression in TNBC based on the best cut results of survival analysis (|logfc| > 0.5, adjust P < 0.05). Our results showed that there were 294 up-regulated SDEGs in high CDKN2A expression groups, which were associated with the positive regulation of transforming growth factor beta receptor signaling pathway, cellular response to metal ion, regulation of actin cytoskeleton, signaling by Rho GTPases, Miro GTPases and RHOBTb3, etc (**Figure 4G**). And 119 down-regulated SDEGs indicated in low CDKN2A expression group correlated with the regulation of production of molecular mediator of the immune response, mitochondrion organization and cytokine signaling in the immune system, etc (**Figure 4G**). The above enriched functional pathways may be the reason for the significant difference in survival between the two groups of TNBC patients. Further, we tried to explore the potential interplay between CDKN2A and TME implicated in TNBC. The IPS score was used to assess the impact of CDKN2A expression on TNBC immunity. The results showed that the low CDKN2A expression was positively correlated with the decreased IPS (**Figure 4F**), which indicates that low expression of CDKN2A might be unresponsible for immunotherapy, probably linking to inhibition of T cell infiltration and suppression of immunogenicity (**Supplementary Figure S5B**). GSE173839 further effectively verified that high expression of CDKN2A had a better immunotherapy response (**Figures 4I, J**). Taken together, our analyses suggested that CDKN2A might influence the progression and prognosis of TNBC and affect the effectiveness of immunotherapy in TNBC through TME, implying the potential of CDKN2A as a pioneering prognostic predictor for TNBC.

### The CDKN2A-derived prognostic model by machine learning for TNBC patients

**Figure 5:**
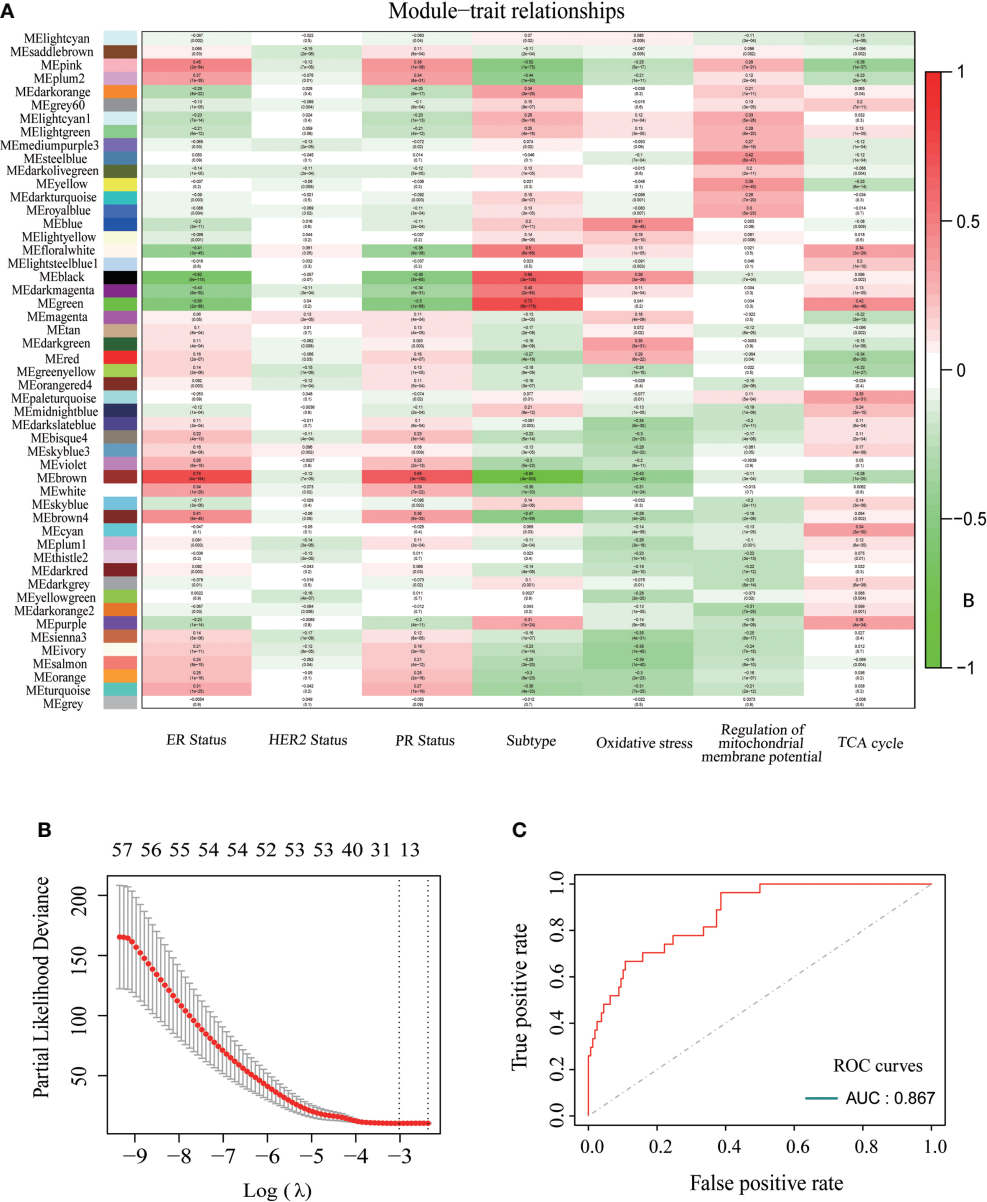
The construction of CDKN2A-derived prognostic model of TNBC. **(A)** The relationships between each module and ER status, HER status, PR status, BRCA subtypes, oxidative stress, regulation of mitochondrial membrane potential and TCA cycle. **(B)** 21 modeling genes determined by lasso algorithm. **(C)** The ROC curves and AUC value of CDKN2A-derived model.

To further explore the relationship between CDKN2A, FAC, and TNBC, on the basis of TCGA-BRCA cohort, a co-expression network and modules of differentially expressed CDKN2A-derived genes were constructed *via* the WGCNA. Overall, the brown and green module had the strongest correlation with TNBC *via* the Kruskal-Wallis test and Tuckey’s honestly significant difference, and simultaneously possessed the most outstanding connection with the FAC activity (**Figure 5A**). A total of 1,924 CDKN2A-derived genes in these two modules were selected for further study. Subsequently, the univariate Cox regression analysis was conducted to gain 106 genes associated with prognosis (**Table 2**). Then, LASSO regression further screened out 21 prognostic genes for constructing the risk predictive model (**Table 3**, **Figure 5B**). On the foundation of 21 genes, the formula of risk scores is as follows:


$$\begin{array}{l} Risk\;Score=AC131097.2\;expression\times 0.603906061605295+TRIM59\;expression\\ \times \left(-0.250311439654858\right)+GRIA1\;expression\;\times 4.75432106611303\\ +EXO1\;expression\times \left(-0.151147624875987\right)+RAPGEF3\;expression\;\\ \times 0.0694637045689866+FAM72C\;expression\times 0.687109193015145\\ +SEPT3\;expression\times \left(-0.15730852851611\right)+FAM228B\;expression\\ \times 0.0245662769232489+AGBL2\;expression\times \left(-0.255478956488232\right)\\ +AGR2\;expression\times \left(-0.0563974779408671\right)+CFAP99\;expression\\ \times 7.67515956697156+CFAP300\;expression\times 0.271605260819289\\ +LYPD6B\;expression\times \left(-0.0224796251790447\right)+NCCRP1\;expression\\ \times 0.0265870429310081+NT5DC2\;expression\times 0.196412885888264\\ +AK8\;expression\times 0.224051779836318+CFAP45\;expression\\ \times 0.226574196279908+ZNF587B\;expression\\ \times \left(-0.00393169189489084\right)+ZNF703\;expression\\ \times 0.213684395875983+LRRC46\;expression\times 0.202045367075346\\ +EMARD\;expression\times 0.0329920921209145. \end{array}$$

**Table 2:** The 106 prognostic genes obtained by the univariate Cox regression analysis.

	gene	HR	z	P value
1	PIGA	0.397277864	-2.198591033	0.027907015
2	PDK1	0.357375189	-2.671129231	0.007559654
3	DLGAP5	0.567091502	-2.062635139	0.039147307
4	ASF1A	0.359053378	-2.653642374	0.007962817
5	ST6GALNAC6	1.898724967	2.019711022	0.043413371
6	SUMO2	0.346718485	-2.365117868	0.018024333
7	BRIP1	0.453575362	-2.119749975	0.034027136
8	AC131097.2	3.937831563	2.632738899	0.008469943
9	CENPF	0.586505608	-2.30669878	0.021071618
10	PTPN2	0.378693961	-2.177849625	0.029417234
11	CHEK2	0.422240769	-2.07745616	0.037759477
12	PAK1IP1	0.483556649	-2.245128753	0.024759868
13	NUS1	0.295640654	-2.890349779	0.003848134
14	C15ORF59	2.956081195	2.237525193	0.025252035
15	GTSE1	0.451487549	-2.438868384	0.014733333
16	TRIM59	0.310058943	-3.060009496	0.0022133
17	FAM111B	0.59996083	-2.269372812	0.023245664
18	ASPM	0.518452041	-2.349979602	0.01877444
19	MCM6	0.523212965	-2.251137167	0.024376851
20	TOM1L2	2.161448416	2.143470398	0.032075346
21	NEIL3	0.462511653	-2.160366585	0.030744302
22	HELLS	0.321344977	-2.789331014	0.005281705
23	ZDHHC1	2.628482692	2.440752437	0.014656698
24	GJC3	1.44465959	2.114886157	0.034439651
25	E2F8	0.425974425	-2.553875056	0.010653148
26	GRIA1	1723.544976	3.621990675	0.000292345
27	KIF11	0.55847202	-2.247271125	0.024622705
28	EXO1	0.460937898	-3.030924746	0.00243806
29	EZH2	0.572485292	-1.983570228	0.047303771
30	YES1	0.601630765	-2.039299095	0.041420186
31	FOXM1	0.650871311	-2.133764473	0.032862065
32	TYMS	0.628826297	-2.080108988	0.037515537
33	RAD51AP1	0.60276431	-2.043099948	0.041042545
34	CENPU	0.493079906	-2.724145067	0.006446818
35	RAPGEF3	3.967600937	2.640190769	0.008285937
36	DUSP4	0.565981807	-2.369869937	0.017794344
37	CENPQ	0.447055144	-2.271846928	0.023095757
38	ZNF883	1.55594112	2.089772472	0.036638243
39	LRRC8D	0.605125564	-2.132304254	0.032981843
40	CNIH2	0.65503232	-2.144605916	0.031984369
41	CEP55	0.562523781	-2.39416595	0.01665821
42	CCDC160	0.325162054	-2.138272672	0.032494619
43	KIF14	0.510799562	-2.092932584	0.036355173
44	ZWILCH	0.445430957	-2.106543951	0.03515713
45	FAM219A	1.893507046	1.964846926	0.049431957
46	KIF18A	0.449222812	-2.402609976	0.016278539
47	TMPO	0.553419185	-2.117824503	0.034189933
48	NFIA	0.638052834	-2.189504267	0.028560209
49	TPCN1	3.290932788	2.415741287	0.015703214
50	RHNO1	0.514295412	-2.204128211	0.027515329
51	CTSF	1.472449338	2.072960013	0.038176001
52	FAM72C	3.066644433	2.822432329	0.004766088
53	SEPT3	0.55369899	-3.504183774	0.000458009
54	APBA2	1.714725532	2.073801231	0.038097775
55	FUT8	0.474158219	-2.111769715	0.034706206
56	LRGUK	0.062432238	-2.110568263	0.034809438
57	ADCY6	2.067368208	2.081207676	0.037414901
58	VWA2	0.497605963	-2.143156172	0.03210056
59	TTC39C	0.508246458	-2.015928299	0.043807474
60	CYB5D2	2.662637199	2.878174729	0.003999835
61	EXOC6	0.291966726	-2.558775588	0.010504153
62	FAM228B	4.003772856	2.542647066	0.011001629
63	FYB2	0.004631401	-1.992285256	0.046339768
64	SPACA9	2.189328115	2.467635909	0.013600858
65	ARNT2	0.680364619	-2.106494914	0.035161384
66	KRT37	20.62888681	2.558603819	0.010509343
67	AGBL2	0.009647128	-2.17119562	0.029916388
68	AGR2	0.591598511	-2.312899108	0.020728187
69	CCNG2	0.505625171	-2.100827065	0.03565615
70	DNAH5	2.989128412	1.980489126	0.047648594
71	CFAP99	9925778.946	3.182566191	0.001459761
72	C16ORF71	3.729277111	2.109173849	0.034929578
73	FOLH1	0.560549119	-2.027960378	0.042564292
74	C11ORF70	2.694865042	2.529973668	0.011407109
75	LYPD6B	0.427966493	-2.203336375	0.027571049
76	TEX9	0.227190102	-2.109559499	0.034896316
77	NCCRP1	1.287452778	2.89136402	0.003835735
78	SLC1A4	0.569212551	-2.344642453	0.019045334
79	PSD3	0.467450303	-2.303773658	0.021235353
80	KITLG	0.589293262	-2.217628814	0.026580152
81	NT5DC2	1.82691064	2.549310104	0.010793628
82	HMGCL	2.643566798	2.3086158	0.02096491
83	AK8	5.224451486	3.075228928	0.00210341
84	TRERF1	0.510437127	-2.029071436	0.042451015
85	PLPPR3	1.450251013	2.269292578	0.02325054
86	PER2	0.403209642	-2.331594507	0.019722033
87	CFAP45	3.034070634	3.486607251	0.000489189
88	TRIM3	2.974918176	2.62253278	0.008727887
89	ZNF587B	0.19192526	-3.234985541	0.001216489
90	KIAA0040	0.620949694	-2.094736682	0.036194406
91	KCNK6	1.592776419	2.606639009	0.00914357
92	ZNF92	0.359485724	-2.793031961	0.005221653
93	PATZ1	0.401765187	-2.695232839	0.007033946
94	FRY	0.338592705	-2.016077604	0.043791862
95	RHOB	1.713754168	2.231692989	0.025635261
96	ZNF586	0.383365946	-2.064031138	0.039014764
97	ZNF703	1.9295092	2.970538785	0.002972779
98	AC008560.1	0.217653792	-2.008129249	0.044629559
99	LRRC46	8.348516913	2.84673037	0.004417076
100	ERMARD	3.155353	2.806137256	0.005013933
101	IKBKB	1.812822308	1.965508385	0.049355426
102	OSCP1	2.410070524	2.698798257	0.006959035
103	AC096887.1	16.86023895	2.189770024	0.02854092
104	INAVA	0.647831638	-2.038272415	0.041522697
105	CASD1	0.407790687	-2.164321144	0.030439711
106	ST8SIA6	0.494039492	-2.094576191	0.036208684

**Table 3:** The 21 prognostic genes for constructing the risk predictive model.

Symbol	Name	Category	Ensembl Version	Description and Functional Summary
**TRIM59**	Tripartite Motif Containing 59	Protein Coding	ENSG00000213186	Activating ubiquitin protein ligase and Acting upstream of or within negative regulation of I-kappaB kinase/NF-kappaB signaling.
**GRIA1**	Glutamate Ionotropic Receptor AMPA Type Subunit 1	Protein Coding	ENSG00000155511	Ionotropic glutamate receptor. This gene belongs to a family of alpha-amino-3-hydroxy-5-methyl-4-isoxazole propionate (AMPA) receptors. It can alternatively splice transcript variants encoding different isoforms.
**EXO1**	Exonuclease 1	Protein Coding	ENSG00000174371	Encoding a protein with 5’ to 3’ exonuclease activity and being essential for male and female meiosis.
**RAPGEF3**	Rap Guanine Nucleotide Exchange Factor 3	Protein Coding	ENSG00000079337	Enabling guanyl-nucleotide exchange factor activity and protein domain specific binding activity.
**FAM72C**	Family With Sequence Similarity 72 Member C	Protein Coding	ENSG00000263513	A neuronal progenitor cell (NPC) self-renewal supporting protein expressed under physiological conditions at low levels in other tissues.
**SEPT3**	Neuronal-specific septin-3	Protein coding	ENSG00000224883	Playing a role in cytokinesis.
**FAM228B**	Family With Sequence Similarity 228 Member B	Protein Coding	ENSG00000219626	FAM228B is a Protein Coding gene. An important paralog of this gene is ENSG00000276087.
**AGBL2**	AGBL Carboxypeptidase 2	Protein Coding	ENSG00000165923	Enabling metallocarboxypeptidase activity and involved in protein side chain deglutamylation.
**AGR2**	Anterior Gradient 2, Protein Disulphide Isomerase Family Member	Protein Coding	ENSG00000106541	Encoding a member of the disulfide isomerase (PDI) family of endoplasmic reticulum proteins that catalyze protein folding and thiol-disulfide interchange reactions.
**CFAP99**	Cilia And Flagella Associated Protein 99	Protein Coding	ENSG00000206113	Predicted to be located in motile cilium.
**CFAP300**	Cilia and Flagella-associated Protein 300	Protein Coding	ENSG00000137691.13	Playing a role in axonemal structure organization and motility.
**LYPD6B**	LY6/PLAUR Domain Containing 6B	Protein Coding	ENSG00000150556	Enabling acetylcholine receptor regulator activity and predicted to be located in extracellular region and plasma membrane.
**NCCRP1**	NCCRP1, F-Box Associated Domain Containing	Protein Coding	ENSG00000188505	Predicted to contribute to ubiquitin protein ligase activity and be involved in positive regulation of cell population proliferation.
**NT5DC2**	5’-Nucleotidase Domain Containing 2	Protein Coding	ENSG00000168268	Predicted to enable 5’-nucleotidase activity and be involved in dephosphorylation.
**AK8**	Adenylate Kinase 8	Protein Coding	ENSG00000165695	Enabling AMP binding activity and nucleobase-containing compound kinase activity.
**CFAP45**	Cilia And Flagella Associated Protein 45	Protein Coding	ENSG00000213085	Enabling AMP binding activity and involved in establishment of left/right asymmetry and flagellated sperm motility.
**ZNF587B**	Zinc Finger Protein 587B	Protein Coding	ENSG00000269343	Enabling DNA-binding transcription repressor activity, RNA polymerase II-specific and RNA polymerase II transcription regulatory region sequence-specific DNA binding activity.
**ZNF703**	Zinc Finger Protein 703	Protein Coding	ENSG00000183779	Enabling DNA-binding transcription factor binding activity.
**LRRC46**	Leucine Rich Repeat Containing 46	Protein Coding	ENSG00000141294	LRRC46 is a Protein Coding gene. Diseases associated with LRRC46 include Ciliary Dyskinesia, Primary, 13. An important paralog of this gene is LRGUK.
**EMARD**	Not Available	Not Available	Not Available	Not Available
**AC131097.2**	Not Available	Not Available	Not Available	Not Available

**Figure 6:**
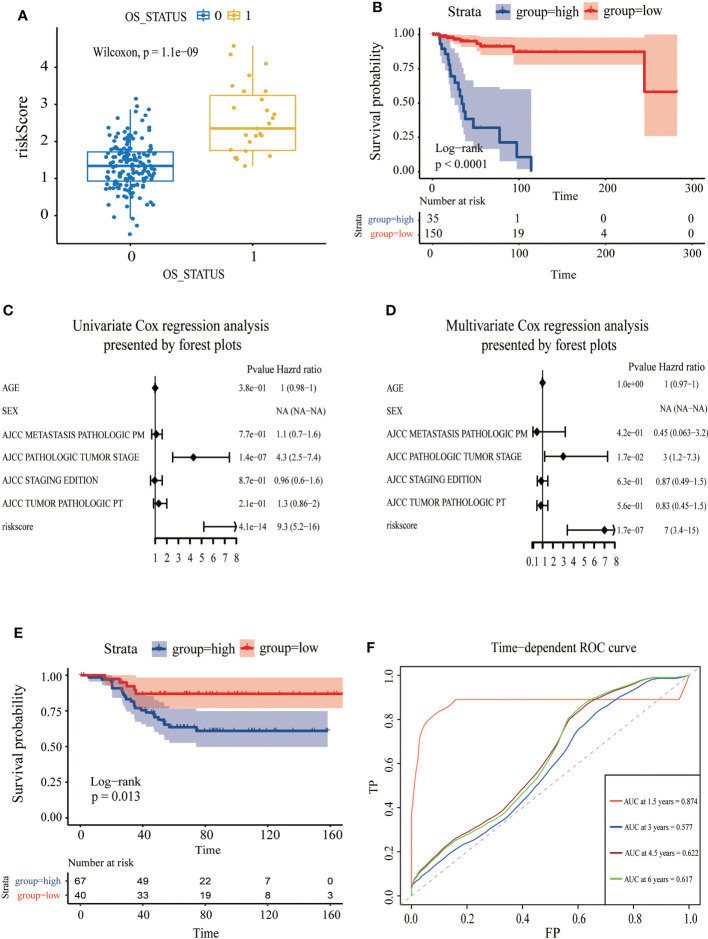
Assessment of the independent prognostic value and validation of CDKN2A-derived prognostic model of TNBC. **(A)** The correlation between OS status and risk score. **(B)** The survival curve of high and low risk score in TNBC. **(C)** Univariate Cox regression analysis of CDKN2A-derived prognostic model. **(D)** Multivariate Cox regression analysis of CDKN2A-derived prognostic model. **(E)** The survival curve verified by the external validation set. **(F)** The time-dependent ROC curve and AUC values respectively at 1.5 years, 3 years, 4.5 years, and 6 years verified by an external validation set.

The AUC was 0.867 and the survival analysis indicated that TNBC patients with a high-risk score possessed a prognosis with misery than those with a low-risk score (P < 0.0001) (**Figures 5C**, **6A, B**). Additionally, univariate and multivariate Cox regression analyses were both used to assess whether the 21 CDKN2A-derived genes signature was an independent prognostic factor for other features, including age, sex, metastasis status, tumor stage, and so on. As the forest plots shown, univariate and multivariate Cox regression analyses both indicated that risk score, age, sex, metastasis status, tumor stage, and pathological status were the independent prognostic factors (**Figures 6C, D**). All results indicated that the 21 CDKN2A-derived genes signature was an independent prognostic factor for TNBC patients.

To further assess the robustness of the CDKN2A-derived genomic model, an independent GEO dataset was used for validation. Reassembly, our scoring system indicated that TNBC patients in the low-risk subgroup had better survival than those in the high-risk subgroup (P = 0.013) (**Figure 6E**). The AUC for OS was 0.874 at 1.5 years, 0.577 at 3 months, 0.622 at 4.5 years, and 0.617 at 6 years in the GSE58812 cohort (**Figure 6F**).

Moreover, 46 TNBC-specific differentially expressed genes (TDEGs) were screened by two subpopulations comparison in TCGA training data, and then the enrichment analysis of TDEGs was conducted. GO functional annotations described those TDEGs mainly involved in response to interferon-gamma, virus receptor binding, several chemokines receptor binding, and so on (**Supplementary Figure S6A**). The analysis of the KEGG pathway revealed enrichment of COVID-19, pertussis, Kaposi sarcoma-associated herpesvirus infection, IL-17 signaling pathway, staphylococcus aureus infection, and so on (**Supplementary Figure S6B**). Additionally, our PPI network also exhibited the correlation between CDKN2A and the genes constructing the model (**Supplementary Figure S6C**).

### Potential therapeutic agents for TNBC patients based on the CDKN2A-derived model

**Figure 7:**
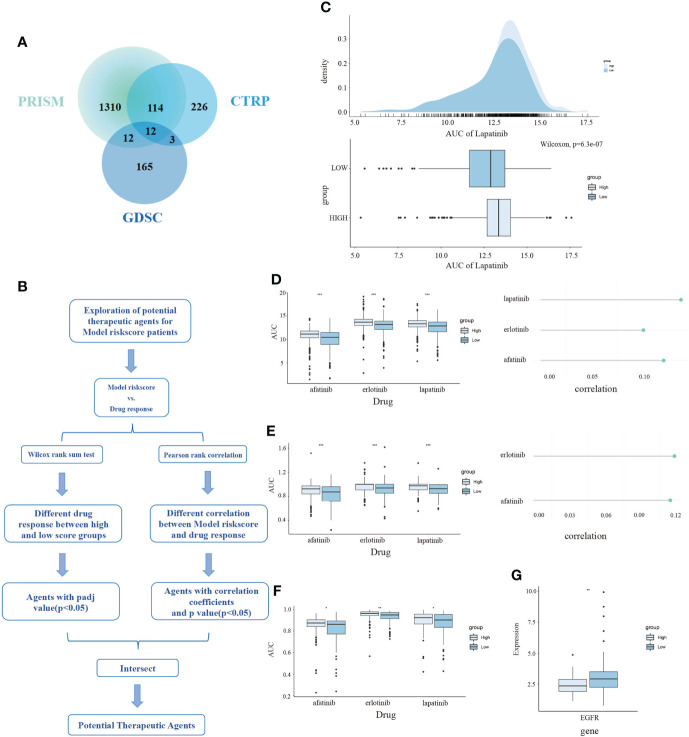
The exploration of potential targeted drugs based on the CDKN2A-derived model for TNBC patients. **(A)** The shared drug between PRISM, GDSC and CTRP. **(B)** The flow chart of exploring potential therapeutic agents. **(C)** The AUC of lapatinib in high and low risk score subpopulations of TNBC patients. **(D–F)** The AUC of three selected drugs in high and low risk score subpopulations of TNBC patients. **(G)** The relationship between AUC values and targets of three drugs. The P values of the figure are shown as follows: *P < 0.05. **P < 0.01. ***P < 0.001.

Profiles of gene expression and drug sensitivity were obtained from the PRISM, CTRP, and GDSC dataset, which was used to build the predictive signature of drug response for TNBC. We obtained a total of 1995 drugs from the three databases, as well as 12 compounds shared among 3 datasets (**Figure 7A**). After removing the drugs whose missing AUC value exceeded 80% and was regarded as NA value, we obtained 174 drugs and 270 cell lines in GDSC, 355 drugs and 638 cell lines in CTRP, as well as 1444 drugs and 462 cell lines in PRISM. The procedure in detail is shown in (**Figure 7B**). We separated the TNBC patients into high and low risk-score subpopulations pursuant to the CDKN2A-derived prognostic model. The difference in AUC estimates of lapatinib was compared *via* the Wilcoxon rank-sum test. Our data demonstrated that the high risk-score group had higher AUC estimates (**Figure 7C**). After confirming the reliability of the calculation method, we made some modifications to the analysis of Yang et al. (44). In our study, we started with the differential drug response analysis between low risk-score group and high risk-score group by the median split. Next, agents with correlation coefficients (P < 0.05) were identified according to Pearson rank correlation analysis between the risk-score of the CDKN2A-derived model and AUC values. Three overlapping drugs were eventually found in these three databases, including afatinib, erlotinib and lapatinib (**Figures 7D–F**). Then, we analyzed the target gene expression difference of three mentioned-above potential drugs between high risk-score and low risk-score subpopulations. Notably, EGFR is the co-target gene of the three candidate drugs. The expression level of EGFR was significantly upregulated in the low risk-score subpopulation (**Figure 7G**). In summary, our outcomes indicated that afatinib, erlotinib and lapatinib could be designated as the potential drugs for low risk-score TNBC patients by targeting EGFR.

## Discussion

The rapidly increasing number of diagnosed BRCA patients results in the urgent need for new biomarkers that can elucidate breast carcinogenesis and predict the immune prognosis (45). In view of problems, such as small sample size, multiple BRCA subtypes, and complex mechanisms of BRCA, previous studies disputed that heterogeneity existed in the expression of CDKN2A in BRCA (46–48) and did not obtain the final verdict of CDKN2A’s effects on BRCA.

The landscape analysis based on the multiple data indicated that CDKN2A had an overexpression and critical values of prognosis in BRCA, hinting at its clinical property as a prognostic biomarker. Additionally, its upregulation was strikingly correlated to DNA hypermethylation. Genetic and epigenetic alterations are both involved in the procession of breast carcinogenesis. The promoter hypermethylation level is commonly associated with transcriptional gene silencing (49). Our results were indicative that DNA hypermethylation of CDKN2A promoted breast carcinogenesis and had a significant association with subtypes of BRCA. Especially in the Luminal and TNBC subtypes, the hypermethylation of CDKN2A was more significant (P < 0.05). Lubecka et al. (50) indicated that the administration of sulforaphane and clofarabine could inhibit the tumor cell growth in breast tissues *via* reactivating methylation-silenced CDKN2A. Thus, inhibiting the hypermethylation levels of CDKN2A could be a potential therapeutic method of BRCA, especially for patients with Luminal and TNBC subtypes. In view of the uncommon phenomenon that CDKN2A showed hypermethylation in BRCA but exhibited high expression, we searched more literatures. Smith et al. (51) reviewed several cases about promoter DNA hypermethylation promoting target gene transcription and they postulated a context-dependent model whereby epigenetic contributions to transcriptional regulation occur in a more complex and dynamic manner, which needs further investigation. The analysis of CDKN2A and drug sensitivity in BRCA expanded clinical applications of CDKN2A. A case-control study (52) indicated the risk of BRCA had a 1.82-fold increase in women with high sensitivity to bleomycin, which reversely confirms our results that CDKN2A upregulation could reduce sensitivity to bleomycin, resulting in a positive prognosis. Herein, regulating the expression of CDKN2A might alter the drug sensitivity and affect the therapeutic results.

As numerous evidence robustly supported, the overload of copper is thought to induce neurotoxicity in neurodegenerative disorders (Parkinson’s disease and Alzheimer’s disease) and hepatocerebral (Wilson’s disease) over several decades (53). However, the previous regulation of ferroptosis to trigger tumor cell death (54) gave us inspiring hints that it is highly viable to regulate the certain copper levels in a suitable concentration to induce the cuproptosis and tumor cell death (55). Ferroptosis indicates an oxidative cell death resulting from the deterioration of antioxidant function and accretion of lipid reactive oxygen species (9). Excess copper can trigger proteotoxic stress and death in cells through the combination with lipoylated components of the tricarboxylic acid (TCA) cycle (8). The stimulation of inflammatory conditions will lead to elevated serum copper levels and trigger oxidative stress, thereby activating the inflammatory response (56). In reverse, inflammation could also accelerate the cytotoxicity mediated by copper *via* overexpressing six-transmembrane epithelial antigens of prostate 4 (STEAP4) (29). Disulfiram/copper was investigated to induce cytotoxic and anti-tumor effects on nasopharyngeal carcinoma cells through p53-mediated ferroptosis and ROS/MAPK pathways (30). Fatty acids degradation can tremendously alter the microbial sensitivity to copper, thus induce copper toxicity (28). Copper also could trigger the expression of GPER, VEGF, and HIF-1α *via* activating EGFR/ERK/c-fos transduction pathway, affecting the angiogenesis and tumor progression in BRCA and LIHC (31). Our study groundbreakingly and vicariously evaluated the activities of ferroptosis and cuproptosis for patients with BRCA based on the seven above-mentioned pathways. Our three subtypes obtained from unsupervised cluster analysis not only exhibited distinct activities in multiple tumor-related pathways but also had critical significance in scores of FAC. Ke et al. (57) indicated that FTL could function as a prognostic and diagnostic ferroptosis regulator in hepatocellular carcinoma *via* random forest analysis, which was consistent with our results that the FTL-dominant cluster possessed a strong connection with ferroptosis. Their resemble conclusion that higher infiltrating immune cells, including Gamma delta T cells and activated CD8+ T cells, emerged in the high FTL expression group, was also confirmed in our study. Hence, regulating pathways involved in CDKN2A-associated genes or designing novel metal-based anticancer agents to induce ferroptosis and cuproptosis may guide us to develop new anti-cancer treatment strategies for BRCA, especially for the patients in the FTL-dominant subtype.

As previous studies reported, the dynamical characteristics of the TME, chemokines, immune checkpoints, and tumor immune infiltration have a clear underlying role in tumorigenesis and progression (58, 59). Surgery, endocrine therapy, and chemotherapy remain the fundamental cornerstones of BRCA, nevertheless, immunotherapy has gradually become one of the neoadjuvant combination therapy strategies (60). Our further analysis proved that CDKN2A overexpression was correlated to the increased immune cells, enhanced immune checkpoints, and elevated chemokines, indicating that CDKN2A might be applied as a potential immunotherapeutic therapy. The profile from CIBERTSORT in our unsupervised groups is highly in line with current studies on immune cell infiltration. Group 1, namely the “cold immune subtype”, showed relatively high levels of naïve B cells, resting memory CD4+ T, and M2 macrophages. Gunderson et al. (22) reported that patients with overexpression of naïve B cells had a sign of misery prognosis, verifying its carcinogenic effect. The higher ratio of resting memory CD4+ T cells in our cold immune subtype was consistent with the hints that resting memory CD4+ T cells predicted an undesirable clinical outcome (61). As an anti-inflammatory and pro-tumor factor, M2 macrophage, was widely recognized as a promoter of metastatic progression and poor prognosis in BRCA (62). However, Spear et al. demonstrated that the infiltration of memory B cells could serve as an immunostimulatory factor and supported the adaptive antitumor immunotherapy (63), which was consistent with our analyses of INF-γ activated subtype. As was mentioned above, our unsupervised groups were correlated with the TME and gave us potential immune therapeutic opportunities by respectively modulating corresponding immune cells in each group.

Additionally, our results from unsupervised clusters analysis were consistent with prior investigations that TNBC was more likely to harbor immunogenicity and more suitable for immunotherapy than other molecular subtypes (64). Moreover, current clinical investigations are paying attention to making non-responders convert to responders or deepening those occurred responses. The previous study reported that the loss of CDKN2A significantly made non-small cell lung cancer patients experience disease progression after immune checkpoint blockade therapy (65). Horn et al. also demonstrated that the frequent loss of the CDKN2A could trigger the susceptibility to IFN-γ resistance *via* JAK2 gene deletion in melanoma (66), which was in line with our conclusion that high expression of CDKN2A potentially benefited from immunotherapy. However, the immunotherapy response of CDKN2A in TNBC has not been reported. Our IPS and verification of external BRCA cohorts (GSE173839) comprehensively suggested that the expression of CDKN2A could modulate the response to immunotherapy to TNBC, and TNBC with high CDKN2A expression patients have higher immunogenicity and benefit from immunotherapy.

Because the overexpression of CDKN2A was indicative of desirable clinical outcomes for TNBC patients, we further conducted WGCNA analysis to determine CDKN2A-derived genes that were chiefly associated with TNBC and pathways of FAC. Determining genes and utilizing cox and lasso analysis, we established a CDKN2A-derived prognostic model, consisting of TRIM59, EXO1, AGR2, ZNF703, and other 17 genes. According to immunohistochemistry, Liu et al. (67) found that TRIM59 levels were notably higher in the TNBC subtype and promoted the malignant behavior *via* regulating the AKT pathway, leading to the undesirable prognosis. Previously, RT-qPCR also proved the overexpression of EXO1 in BRCA cells MDA-MB231, and the elevated EXO1 might be utilized as an indicator of poor BRCA prognosis (68). A clinical observation study (69) *via* the cross-sectional method indicated that AGR2 expression is positively associated with the incidence of distant metastases in BRCA and upregulated AGR2 was a poor prognosis predictor. Current research reported that ZNF703 expressed in approximately 34.2% of TNBC *via* immunohistochemistry and the knockdown of ZNF703 triggered a powerful inhibition of TNBC cell proliferation and cell cycle, along with the downregulation of cyclin D1, CDK4, CDK6, and E2F1 (70). Remaining genes were firstly explored to have effects on the prognosis of TNBC patients. Deeper studies of the biological roles of these genes in TNBC are warranted and clinical investigations of this signature need to be further tested.

In terms of the high heterogeneity of TNBC, it’s incredibly difficult to find new curative targets and develop novel targeted therapy. DNA microarray analysis conducted by Nielsen et al. (71) indicated that overexpression of EGFR existed in 60% of TNBC samples, which was consistent with our results. The study of Livasy et al. (72) also validified that approximately 70% of TNBC samples significantly expressed elevated EGFR. Hence, it is inferred that EGFR may be a promising curative target in TNBC, especially for TNBC patients with low risk-score according to our model. As the irreversible ErbB family blocker, afatinib (AFT) was approved by the FDA to treat the advanced EGFR mutation-positive NSCLC (73). The investigations of AFT treatment in BRCA are undergoing. In an open-label, multicenter, and phase II clinical trial, Hickish et al. (74) reported that for metastatic BRCA patients whose prior HER2-targeted therapy had undesirably failed, AFT alone and combined with paclitaxel or vinorelbine could enhance the objective response. Our data demonstrated AFT may have a good therapeutic effect on TNBC. Coherent with our outcomes, Wang et al. (75) developed AFT/2-BP@PLGA@MD, a poly(d,l-lactide-glycolide) (PLGA)-based intelligent bionic nanoplatform, which was covered under a cancer cell membrane to block PD-1 and PD-L1. AFT/2-BP@PLGA@MD nanoparticles integrated the targeted therapy of AFT and immunotherapy, exhibiting enhanced inhibition of the growth of TNBC. As a dual inhibitor of EGFR and HER2, lapatinib could also induce inhibition of p-Akt and CIP2A and trigger apoptosis in TNBC cell lines (76). LHNPs, human serum albumin nanoparticles loaded with lapatinib, were developed by the advanced nanoparticle albumin-bound technology, and could inhibit the brain metastasis from TNBC ascribed to the downregulation of metastasis-related proteins (77). Collectively, based on the model, we proposed three drugs that may be applicable to TNBC patients with low risk-score. Previous ssGSEA results also presented the CDKN2A-associated genes also correlated to the EGFR activity, indicating that CDKN2A may function as a promising predictive biomarker for anti-EGFR therapy in TNBC. Moreover, drawing support from advanced nanoparticle technology, we put forward the perspective that developing novel nanoparticles combined with immunotherapy and targeted therapy to achieve a better prognosis for TNBC patients.

## Conclusion

In summary, our study comprehensively analyzed the biological role and prognostic values of CDKN2A in BRCA. Given the strong association between CDKN2A and FAC, we indicated that regulating pathways involved in CDKN2A-associated genes or designing novel metal-based anticancer agents to induce ferroptosis and cuproptosis may guide us to develop new anti-cancer treatment strategies. Besides, we substantively found that CDKN2A may serve as the pioneering prognostic predictor for TNBC. TNBC patients with high CDKN2A expression possess the higher immunogenicity and benefit from immunotherapy. The CDKN2A-derived model we established can also guide the prognosis of TNBC patients. To further guide the treatment, we also provided three drugs for precision medicine of TNBC *via* targeting EGFR and indicated that CDKN2A may function as a promising predictive biomarker for anti-EGFR therapy in TNBC. Therefore, this investigation provides a rationale and offers fresh perspectives and orientations for TNBC treatment.

## Data availability statement

The original contributions presented in the study are included in the article/**Supplementary Material**. Further inquiries can be directed to the corresponding authors.

## Author contributions

TC and YW conceived the presented idea and analyzed the data under the supervision of CH. ZL, YY, SS and MG collected the data. TC and YW took the lead in drafting the manuscript with input from all authors. BS revised the manuscript. SS and BS interpreted results from a clinical point of view. All authors read and approved the final manuscript.

## Funding

This work was funded by The Science and Technology Development Fund, Macau SAR 462 (File no. 0020/2021/A, 001/2020/ALC, SKL-QRCM (MUST)-2020-2022), and Dr. Neher’s Biophysics Laboratory for Innovative Drug Discovery, State Key Laboratory of Quality Research in Chinese Medicine, Macau University of Science and Technology, Macau, China (Grant no. FRG-21-032-SKL).

## Conflict of interest

The authors declare that the research was conducted in the absence of any commercial or financial relationships that could be construed as a potential conflict of interest.

## Publisher’s note

All claims expressed in this article are solely those of the authors and do not necessarily represent those of their affiliated organizations, or those of the publisher, the editors and the reviewers. Any product that may be evaluated in this article, or claim that may be made by its manufacturer, is not guaranteed or endorsed by the publisher.

## Glossary

**Table:**

BRCA	breast cancer
BLCA	bladder urothelial carcinoma
CESC	cervical squamous cell carcinoma and endocervical adenocarcinoma
CHOL	cholangiocarcinoma
COAD	colon adenocarcinoma
HNSC	head and neck cancer
KICH	kidney chromophobe
KIRC	kidney renal clear cell carcinoma
KIRP	kidney renal papillary cell carcinoma
LIHC	liver hepatocellular carcinoma
LUAD	lung adenocarcinoma
LUSC	lung squamous cell carcinoma
PRAD	prostate adenocarcinoma
READ	rectum adenocarcinoma
STAD	stomach adenocarcinoma
THCA	thyroid carcinoma
UCEC	uterine corpus endometrial carcinoma
TNBC	triple-negative breast cancer
CDKN2A	cyclin-dependent kinase inhibitor 2A
FAC	ferroptosis and cuproptosis
TME	tumor microenvironment
Her2	human epidermal growth factor receptor 2
ER/PR	estrogen receptor/progesterone receptor
ROS	reactive oxygen species
TCA	tricarboxylic acid
mRNA	the messenger RNA
IPS	Immunophenoscore
MAPK	mitogen-activated protein kinase
VEGF	vascular endothelial growth factor
PCA	principal component analysis
Bicor	biweight midcorrelation
ROC	receiver operating characteristic
GDSC	Genomics of Drug Sensitivity in Cancer
PRISM	Profiling Relative Inhibition Simultaneously in Mixtures
CTRP	Cancer Therapeutics Response Portal
OS	overall survival
DEGs	differentially expressed genes
TDEGs	TNBC-specific differentially expressed genes
STEAP4	six-transmembrane epithelial antigens of prostate 4
GPER	G protein-coupled estrogen receptor
AFT	afatinib
TCGA	The Cancer Genome Atlas
GEO	Gene Expression Omnibus
TIMER	Tumor Immune Estimation Resource
GEPIA	Gene expression profiling interactive analysis
HPA	Human Protein Atlas
TISIDB	Tumor and Immune System Interaction Database
CIBERSORT	Cell-type Identification By Estimating Relative Subsets Of RNA Transcripts
ssGSEA	Single-Sample Gene Set Enrichment Analysis
TCIA	The Cancer Immunome Atlas
GO	Gene Ontology
KEGG	Kyoto Encyclopedia of Genes
Genomes	
MSigDB	Molecular Signatures Database
LASSO	Least Absolute Shrinkage and Selection Operator.

## References

[1] Sung H Ferlay J Siegel RL Laversanne M Soerjomataram I Jemal A. Global cancer statistics 2020: GLOBOCAN estimates of incidence and mortality worldwide for 36 cancers in 185 countries. CA Cancer J Clin (2021) 71(3):209–49. doi: 10.3322/caac.21660 33538338

[2] Dawson SJ Provenzano E Caldas C. Triple negative breast cancers: clinical and prognostic implications. Eur J Cancer. (2009) 45 Suppl 1:27–40. doi: 10.1016/S0959-8049(09)70013-9 19775602

[3] Dent R Trudeau M Pritchard KI Hanna WM Kahn HK Sawka CA. Triple-negative breast cancer: clinical features and patterns of recurrence. Clin Cancer Res (2007) 13(15 Pt 1):4429–34. doi: 10.1158/1078-0432.CCR-06-3045 17671126

[4] Chan SH Chiang J Ngeow J. CDKN2A germline alterations and the relevance of genotype-phenotype associations in cancer predisposition. Hereditary Cancer Clin practice. (2021) 19(1):21. doi: 10.1186/s13053-021-00178-x PMC7992806 33766116

[5] Liggett WH Jr. Sidransky D. Role of the p16 tumor suppressor gene in cancer. J Clin Oncol (1998) 16(3):1197–206. doi: 10.1200/JCO.1998.16.3.1197 9508208

[6] Luan Y Zhang W Xie J Mao J. CDKN2A inhibits cell proliferation and invasion in cervical cancer through LDHA-mediated AKT/mTOR pathway. Clin Trans (2021) 23(2):222–8. doi: 10.1007/s12094-020-02409-4 32594303

[7] Shi J Wu P Sheng L Sun W Zhang H. Ferroptosis-related gene signature predicts the prognosis of papillary thyroid carcinoma. Cancer Cell Int (2021) 21(1):669. doi: 10.1186/s12935-021-02389-7 34906147 PMC8670268

[8] Tsvetkov P Coy S Petrova B Dreishpoon M Verma A Abdusamad M. Copper induces cell death by targeting lipoylated TCA cycle proteins. Science (2022) 375(6586):1254–61. doi: 10.1126/science.abf0529 PMC9273333 35298263

[9] Li J Cao F Yin HL Huang ZJ Lin ZT Mao N. Ferroptosis: past, present and future. Cell Death Dis (2020) 11(2):88. doi: 10.1038/s41419-020-2298-2 32015325 PMC6997353

[10] Chen D Tavana O Chu B Erber L Chen Y Baer R. NRF2 is a major target of ARF in p53-independent tumor suppression. Mol Cell (2017) 68(1):224–32.e4. doi: 10.1016/j.molcel.2017.09.009 28985506 PMC5683418

[11] Aftab A Shahzad S Hussain HMJ Khan R Irum S Tabassum S. CDKN2A/P16INK4A variants association with breast cancer and their in-silico analysis. Breast Cancer. (2019) 26(1):11–28. doi: 10.1007/s12282-018-0894-0 30039340

[12] Li T Fan J Wang B Traugh N Chen Q Liu JS. TIMER: A web server for comprehensive analysis of tumor-infiltrating immune cells. Cancer Res (2017) 77(21):e108–e10. doi: 10.1158/0008-5472.CAN-17-0307 PMC6042652 29092952

[13] Tang Z Li C Kang B Gao G Li C Zhang Z. GEPIA: a web server for cancer and normal gene expression profiling and interactive analyses. Nucleic Acids Res (2017) 45(W1):W98–w102. doi: 10.1093/nar/gkx247 28407145 PMC5570223

[14] Thul PJ Lindskog C. The human protein atlas: A spatial map of the human proteome. Protein Sci Publ Protein Society (2018) 27(1):233–44. doi: 10.1002/pro.3307 PMC5734309 28940711

[15] Chandrashekar DS Bashel B Balasubramanya SAH Creighton CJ Ponce-Rodriguez I Chakravarthi B. UALCAN: A portal for facilitating tumor subgroup gene expression and survival analyses. Neoplasia (New York NY) (2017) 19(8):649–58. doi: 10.1016/j.neo.2017.05.002 PMC5516091 28732212

[16] Koch A De Meyer T Jeschke J Van Criekinge W. MEXPRESS: visualizing expression, DNA methylation and clinical TCGA data. BMC Genomics (2015) 16:636. doi: 10.1186/s12864-015-1847-z 26306699 PMC4549898

[17] Gao J Aksoy BA Dogrusoz U Dresdner G Gross B Sumer SO. Integrative analysis of complex cancer genomics and clinical profiles using the cBioPortal. Sci Signaling (2013) 6(269):pl1. doi: 10.1126/scisignal.2004088 PMC4160307 23550210

[18] Tate JG Bamford S Jubb HC Sondka Z Beare DM Bindal N. COSMIC: the catalogue of somatic mutations in cancer. Nucleic Acids Res (2019) 47(D1):D941–D7. doi: 10.1093/nar/gky1015 PMC6323903 30371878

[19] Ru B Wong CN Tong Y Zhong JY Zhong SSW Wu WC. TISIDB: an integrated repository portal for tumor-immune system interactions. Bioinf (Oxford England) (2019) 35(20):4200–2. doi: 10.1093/bioinformatics/btz210 30903160

[20] Reinhold WC Sunshine M Liu H Varma S Kohn KW Morris J. CellMiner: a web-based suite of genomic and pharmacologic tools to explore transcript and drug patterns in the NCI-60 cell line set. Cancer Res (2012) 72(14):3499–511. doi: 10.1158/0008-5472.CAN-12-1370 PMC3399763 22802077

[21] Newman AM Liu CL Green MR Gentles AJ Feng W Xu Y. Robust enumeration of cell subsets from tissue expression profiles. Nat Methods (2015) 12(5):453–7. doi: 10.1038/nmeth.3337 PMC4739640 25822800

[22] Yoshihara K Shahmoradgoli M Martinez E Vegesna R Kim H Torres-Garcia W. Inferring tumour purity and stromal and immune cell admixture from expression data. Nat Commun (2013) 4:2612. doi: 10.1038/ncomms3612 24113773 PMC3826632

[23] Barbie DA Tamayo P Boehm JS Kim SY Moody SE Dunn IF. Systematic RNA interference reveals that oncogenic KRAS-driven cancers require TBK1. Nature (2009) 462(7269):108–12. doi: 10.1038/nature08460 PMC2783335 19847166

[24] Sokolov A Carlin DE Paull EO Baertsch R Stuart JM. Pathway-based genomics prediction using generalized elastic net. PLoS Comput Biol (2016) 12(3):e1004790. doi: 10.1371/journal.pcbi.1004790 26960204 PMC4784899

[25] Hu J Yu A Othmane B Qiu D Li H Li C. Siglec15 shapes a non-inflamed tumor microenvironment and predicts the molecular subtype in bladder cancer. Theranostics (2021) 11(7):3089–108. doi: 10.7150/thno.53649 PMC7847675 33537076

[26] Subramanian A Tamayo P Mootha VK Mukherjee S Ebert BL Gillette MA. Gene set enrichment analysis: a knowledge-based approach for interpreting genome-wide expression profiles. Proc Natl Acad Sci USA (2005) 102(43):15545–50. doi: 10.1073/pnas.0506580102 PMC1239896 16199517

[27] Charoentong P Finotello F Angelova M Mayer C Efremova M Rieder D. Pan-cancer immunogenomic analyses reveal genotype-immunophenotype relationships and predictors of response to checkpoint blockade. Cell Rep (2017) 18(1):248–62. doi: 10.1016/j.celrep.2016.12.019 28052254

[28] Avery SV Howlett NG Radice S. Copper toxicity towards saccharomyces cerevisiae: dependence on plasma membrane fatty acid composition. Appl Environ Microbiol (1996) 62(11):3960–6. doi: 10.1128/aem.62.11.3960-3966.1996 PMC168214 8899983

[29] Jiang C Wu B Xue M Lin J Hu Z Nie X. Inflammation accelerates copper-mediated cytotoxicity through induction of six-transmembrane epithelial antigens of prostate 4 expression. Immunol Cell Biol (2021) 99(4):392–402. doi: 10.1111/imcb.12427 33179273

[30] Li Y Chen F Chen J Chan S He Y Liu W. Disulfiram/Copper induces antitumor activity against both nasopharyngeal cancer cells and cancer-associated fibroblasts through ROS/MAPK and ferroptosis pathways. Cancers (Basel) (2020) 12(1):138. doi: 10.3390/cancers12010138 PMC7017005 31935835

[31] Rigiracciolo DC Scarpelli A Lappano R Pisano A Santolla MF De Marco P. Copper activates HIF-1alpha/GPER/VEGF signalling in cancer cells. Oncotarget (2015) 6(33):34158–77. doi: 10.18632/oncotarget.5779 PMC4741443 26415222

[32] Zhou Y Zhou B Pache L Chang M Khodabakhshi AH Tanaseichuk O. Metascape provides a biologist-oriented resource for the analysis of systems-level datasets. Nat Commun (2019) 10(1):1523. doi: 10.1038/s41467-019-09234-6 30944313 PMC6447622

[33] Szklarczyk D Gable AL Nastou KC Lyon D Kirsch R Pyysalo S. The STRING database in 2021: customizable protein-protein networks, and functional characterization of user-uploaded gene/measurement sets. Nucleic Acids Res (2021) 49(D1):D605–D12. doi: 10.1093/nar/gkaa10749 PMC7779004 33237311

[34] Langfelder P Horvath S. WGCNA: an r package for weighted correlation network analysis. BMC Bioinf (2008) 9:559. doi: 10.1186/1471-2105-9-559 PMC2631488 19114008

[35] Langfelder P Horvath S. Fast r functions for robust correlations and hierarchical clustering. J Stat Software (2012) 46(11):11. doi: 10.18637/jss.v046.i11 PMC3465711 23050260

[36] Dill CD Dammer EB Griffen TL Seyfried NT Lillard JW Jr. A network approach reveals driver genes associated with survival of patients with triple-negative breast cancer. iScience (2021) 24(5):102451. doi: 10.1016/j.isci.2021.102451 34007962 PMC8111681

[37] Wang H Lengerich BJ Aragam B Xing EP. Precision lasso: accounting for correlations and linear dependencies in high-dimensional genomic data. Bioinformatics (2019) 35(7):1181–7. doi: 10.1093/bioinformatics/bty750 PMC6449749 30184048

[38] Hua X Zhao W Pesatori AC Consonni D Caporaso NE Zhang T. Genetic and epigenetic intratumor heterogeneity impacts prognosis of lung adenocarcinoma. Nat Commun (2020) 11(1):2459. doi: 10.1038/s41467-020-16295-5 32424208 PMC7235245

[39] Yang W Soares J Greninger P Edelman EJ Lightfoot H Forbes S. Genomics of drug sensitivity in cancer (GDSC): a resource for therapeutic biomarker discovery in cancer cells. Nucleic Acids Res (2013) 41(Database issue):D955–61. doi: 10.1093/nar/gks1111 PMC3531057 23180760

[40] Lyons TG. Targeted therapies for triple-negative breast cancer. Curr Treat Options Oncol (2019) 20(11):82. doi: 10.1007/s11864-019-0682-x 31754897

[41] Chen YP Wang YQ Lv JW Li YQ Chua MLK Le QT. Identification and validation of novel microenvironment-based immune molecular subgroups of head and neck squamous cell carcinoma: implications for immunotherapy. Ann Oncol (2019) 30(1):68–75. doi: 10.1093/annonc/mdy470 30407504

[42] Sacher AG St Paul M Paige CJ Ohashi PS. Cytotoxic CD4(+) T cells in bladder cancer-a new license to kill. Cancer Cell (2020) 38(1):28–30. doi: 10.1016/j.ccell.2020.06.013 32663467

[43] Pitt JM Andre F Amigorena S Soria JC Eggermont A Kroemer G. Dendritic cell-derived exosomes for cancer therapy. J Clin Invest (2016) 126(4):1224–32. doi: 10.1172/JCI81137 PMC4811123 27035813

[44] Chu G Shan W Ji X Wang Y Niu H. Multi-omics analysis of novel signature for immunotherapy response and tumor microenvironment regulation patterns in urothelial cancer. Front Cell Dev Biol (2021) 9:764125. doi: 10.3389/fcell.2021.764125 34926452 PMC8678486

[45] Cheng T Chen P Chen J Deng Y Huang C. Landscape analysis of matrix metalloproteinases unveils key prognostic markers for patients with breast cancer. Front Genet (2021) 12:809600. doi: 10.3389/fgene.2021.809600 35069702 PMC8770541

[46] Bartels S van Luttikhuizen JL Christgen M Magel L Luft A Hanzelmann S. CDKN2A loss and PIK3CA mutation in myoepithelial-like metaplastic breast cancer. J Pathol (2018) 245(3):373–83. doi: 10.1002/path.5091 29708279

[47] Witkiewicz AK Rivadeneira DB Ertel A Kline J Hyslop T Schwartz GF. Association of RB/p16-pathway perturbations with DCIS recurrence: dependence on tumor versus tissue microenvironment. Am J Pathol (2011) 179(3):1171–8. doi: 10.1016/j.ajpath.2011.05.043 PMC3157259 21756866

[48] Herschkowitz JI He X Fan C Perou CM. The functional loss of the retinoblastoma tumour suppressor is a common event in basal-like and luminal b breast carcinomas. Breast Cancer Res (2008) 10(5):R75. doi: 10.1186/bcr2142 18782450 PMC2614508

[49] Lewis CM Cler LR Bu DW Zochbauer-Muller S Milchgrub S Naftalis EZ. Promoter hypermethylation in benign breast epithelium in relation to predicted breast cancer risk. Clin Cancer Res (2005) 11(1):166–72. doi: 10.1158/1078-0432.166.11.1 15671542

[50] Lubecka K Kaufman-Szymczyk A Fabianowska-Majewska K. Inhibition of breast cancer cell growth by the combination of clofarabine and sulforaphane involves epigenetically mediated CDKN2A upregulation. Nucleosides Nucleotides Nucleic Acids (2018) 37(5):280–9. doi: 10.1080/15257770.2018.1453075 29634384

[51] Smith J Sen S Weeks RJ Eccles MR Chatterjee A. Promoter DNA hypermethylation and paradoxical gene activation. Trends Cancer (2020) 6(5):392–406. doi: 10.1016/j.trecan.2020.02.007 32348735

[52] Hu M Han D Sun S Yan Y Zhang J Zhou Y. Bleomycin-induced mutagen sensitivity, passive smoking, and risk of breast cancer in Chinese women: a case-control study. Cancer Causes Control (2013) 24(4):629–36. doi: 10.1007/s10552-012-0137-1 23371556

[53] Pal A. Copper toxicity induced hepatocerebral and neurodegenerative diseases: an urgent need for prognostic biomarkers. Neurotoxicology (2014) 40:97–101. doi: 10.1016/j.neuro.2013.12.001 24342654

[54] Liang C Zhang X Yang M Dong X. Recent progress in ferroptosis inducers for cancer therapy. Adv Mater (2019) 31(51):e1904197. doi: 10.1002/adma.201904197 31595562

[55] Ge EJ Bush AI Casini A Cobine PA Cross JR DeNicola GM. Connecting copper and cancer: from transition metal signalling to metalloplasia. Nat Rev Cancer (2022) 22(2):102–13. doi: 10.1038/s41568-021-00417-2 PMC8810673 34764459

[56] Pereira TC Campos MM Bogo MR. Copper toxicology, oxidative stress and inflammation using zebrafish as experimental model. J Appl Toxicol (2016) 36(7):876–85. doi: 10.1002/jat.3303 26888422

[57] Ke S Wang C Su Z Lin S Wu G. Integrated analysis reveals critical ferroptosis regulators and FTL contribute to cancer progression in hepatocellular carcinoma. Front Genet (2022) 13:897683. doi: 10.3389/fgene.2022.897683 35651950 PMC9149379

[58] Neal JT Li X Zhu J Giangarra V Grzeskowiak CL Ju J. Organoid modeling of the tumor immune microenvironment. Cell (2018) 175(7):1972–88 e16. doi: 10.1016/j.cell.2018.11.021 30550791 PMC6656687

[59] Kaderbhai C Tharin Z Ghiringhelli F. The role of molecular profiling to predict the response to immune checkpoint inhibitors in lung cancer. Cancers (Basel). (2019) 11(2):201. doi: 10.3390/cancers11020201 PMC6406957 30744168

[60] Loibl S Poortmans P Morrow M Denkert C Curigliano G. Breast cancer. Lancet (2021) 397(10286):1750–69. doi: 10.1016/S0140-6736(20)32381-3 33812473

[61] Gu J Zhang J Huang W Tao T Huang Y Yang L. Activating miRNA-mRNA network in gemcitabine-resistant pancreatic cancer cell associates with alteration of memory CD4(+) T cells. Ann Transl Med (2020) 8(6):279. doi: 10.21037/atm.2020.03.53 32355723 PMC7186712

[62] Xiao Y Ma D Zhao S Suo C Shi J Xue MZ. Multi-omics profiling reveals distinct microenvironment characterization and suggests immune escape mechanisms of triple-negative breast cancer. Clin Cancer Res (2019) 25(16):5002–14. doi: 10.1158/1078-0432.CCR-18-3524 30837276

[63] Spear S Candido JB McDermott JR Ghirelli C Maniati E Beers SA. Discrepancies in the tumor microenvironment of spontaneous and orthotopic murine models of pancreatic cancer uncover a new immunostimulatory phenotype for b cells. Front Immunol (2019) 10:542. doi: 10.3389/fimmu.2019.00542 30972056 PMC6445859

[64] Emens LA. Breast cancer immunotherapy: Facts and hopes. Clin Cancer Res (2018) 24(3):511–20. doi: 10.1158/1078-0432.CCR-16-3001 PMC5796849 28801472

[65] Gutiontov SI Turchan WT Spurr LF Rouhani SJ Chervin CS Steinhardt G. CDKN2A loss-of-function predicts immunotherapy resistance in non-small cell lung cancer. Sci Rep (2021) 11(1):20059. doi: 10.1038/s41598-021-99524-1 34625620 PMC8501138

[66] Horn S Leonardelli S Sucker A Schadendorf D Griewank KG Paschen A. Tumor CDKN2A-associated JAK2 loss and susceptibility to immunotherapy resistance. J Natl Cancer Inst (2018) 110(6):677–81. doi: 10.1093/jnci/djx271 29917141

[67] Liu Y Dong Y Zhao L Su L Diao K Mi X. TRIM59 overexpression correlates with poor prognosis and contributes to breast cancer progression through AKT signaling pathway. Mol Carcinog (2018) 57(12):1792–802. doi: 10.1002/mc.22897 30175868

[68] Liu J Zhang J. Elevated EXO1 expression is associated with breast carcinogenesis and poor prognosis. Ann Transl Med (2021) 9(2):135. doi: 10.21037/atm-20-7922 33569437 PMC7867906

[69] Kereh DS Pieter J Hamdani W Haryasena H Sampepajung D Prihantono P. Correlation of AGR2 expression with the incidence of metastasis in luminal breast cancer. Breast Dis (2021) 40(S1):S103–S7. doi: 10.3233/BD-219015 34092584

[70] Zhang X Mu X Huang O Wang Z Chen J Chen D. ZNF703 promotes triple-negative breast cancer cells through cell-cycle signaling and associated with poor prognosis. BMC Cancer (2022) 22(1):226. doi: 10.1186/s12885-022-09286-w 35236318 PMC8889678

[71] Nielsen TO Hsu FD Jensen K Cheang M Karaca G Hu Z. Immunohistochemical and clinical characterization of the basal-like subtype of invasive breast carcinoma. Clin Cancer Res (2004) 10(16):5367–74. doi: 10.1158/1078-0432.CCR-04-0220 15328174

[72] Livasy CA Karaca G Nanda R Tretiakova MS Olopade OI Moore DT. Phenotypic evaluation of the basal-like subtype of invasive breast carcinoma. Mod Pathol (2006) 19(2):264–71. doi: 10.1038/modpathol.3800528 16341146

[73] Wecker H Waller CF. Afatinib. Recent Results Cancer Res (2018) 211:199–215. doi: 10.1007/978-3-319-91442-8_14 30069769

[74] Hickish T Mehta A Liu MC Huang CS Arora RS Chang YC. Afatinib alone and in combination with vinorelbine or paclitaxel, in patients with HER2-positive breast cancer who failed or progressed on prior trastuzumab and/or lapatinib (LUX-breast 2): an open-label, multicenter, phase II trial. Breast Cancer Res Treat (2022) 192(3):593–602. doi: 10.1007/s10549-021-06449-4 35138529 PMC8960620

[75] Wang X Zhu X Li B Wei X Chen Y Zhang Y. Intelligent biomimetic nanoplatform for systemic treatment of metastatic triple-negative breast cancer *via* enhanced EGFR-targeted therapy and immunotherapy. ACS Appl Mater Interfaces (2022) 14:23152–63. doi: 10.1021/acsami.2c02925 35549005

[76] Liu CY Hu MH Hsu CJ Huang CT Wang DS Tsai WC. Lapatinib inhibits CIP2A/PP2A/p-akt signaling and induces apoptosis in triple negative breast cancer cells. Oncotarget (2016) 7(8):9135–49. doi: 10.18632/oncotarget.7035 PMC4891031 26824320

[77] Wan X Zheng X Pang X Pang Z Zhao J Zhang Z. Lapatinib-loaded human serum albumin nanoparticles for the prevention and treatment of triple-negative breast cancer metastasis to the brain. Oncotarget (2016) 7(23):34038–51. doi: 10.18632/oncotarget.8697 PMC5085136 27086917

