# Supplementary figures and images for: CDKN2A-mediated molecular subtypes characterize the hallmarks of tumor microenvironment and guide precision medicine in triple-negative breast cancer
Source: Front Immunol. 2022 Aug 16;13:970950. doi: 10.3389/fimmu.2022.970950 (PMC9424905; doi:10.3389/fimmu.2022.970950)

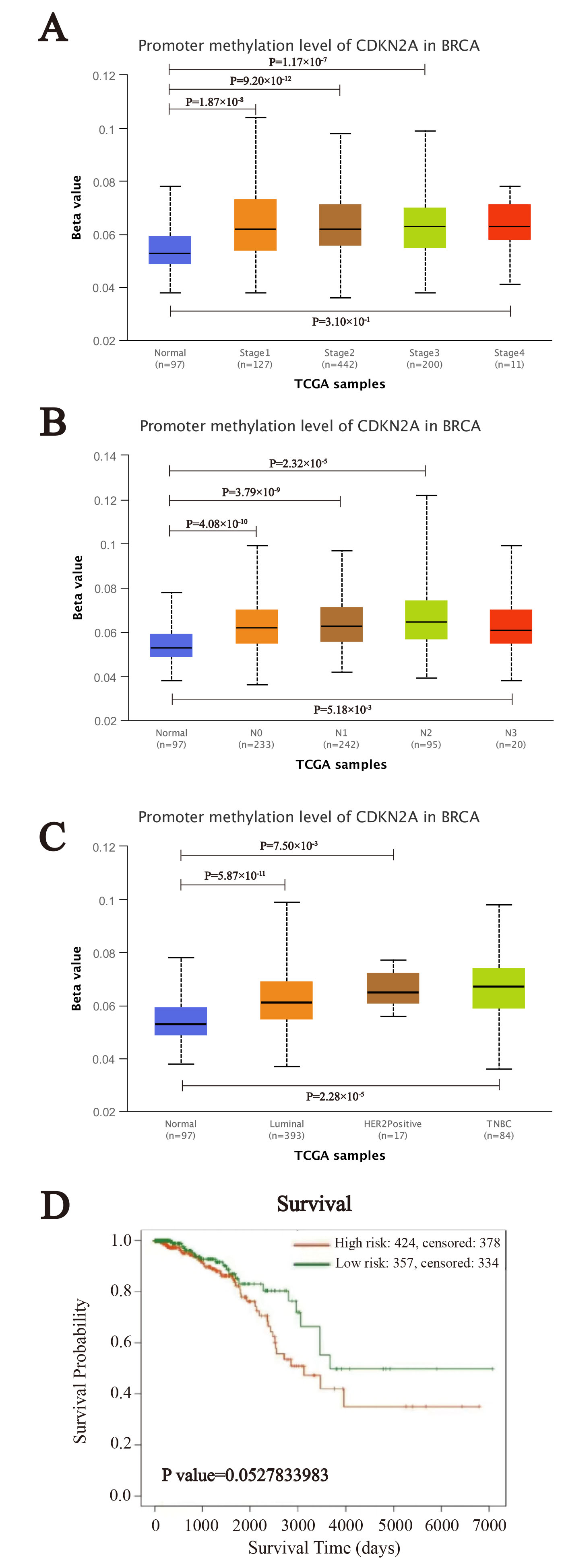

Supplement: Supplementary file 1 [file Image_1.jpeg]

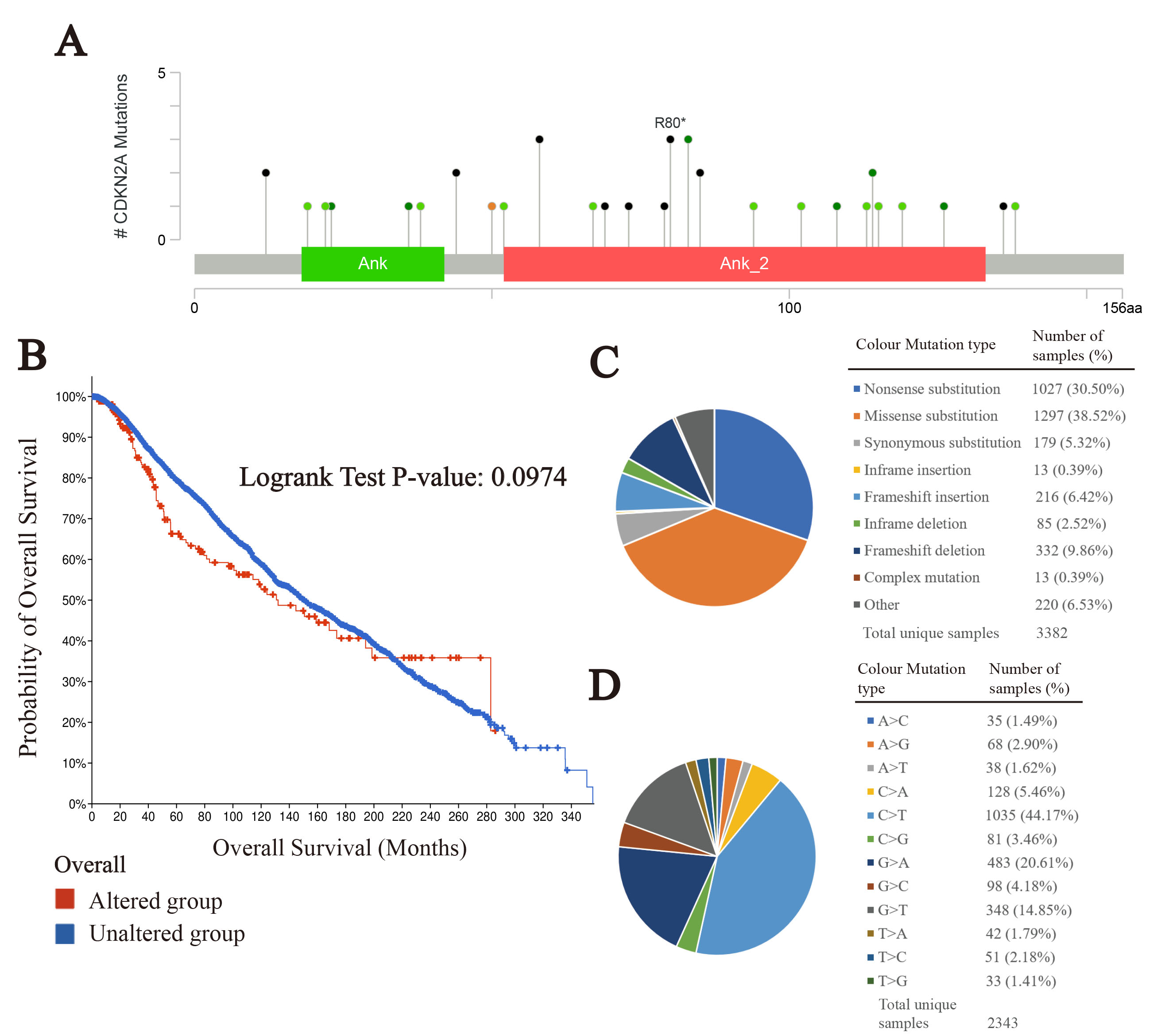

Supplement: Supplementary file 2 [file Image_2.jpeg]

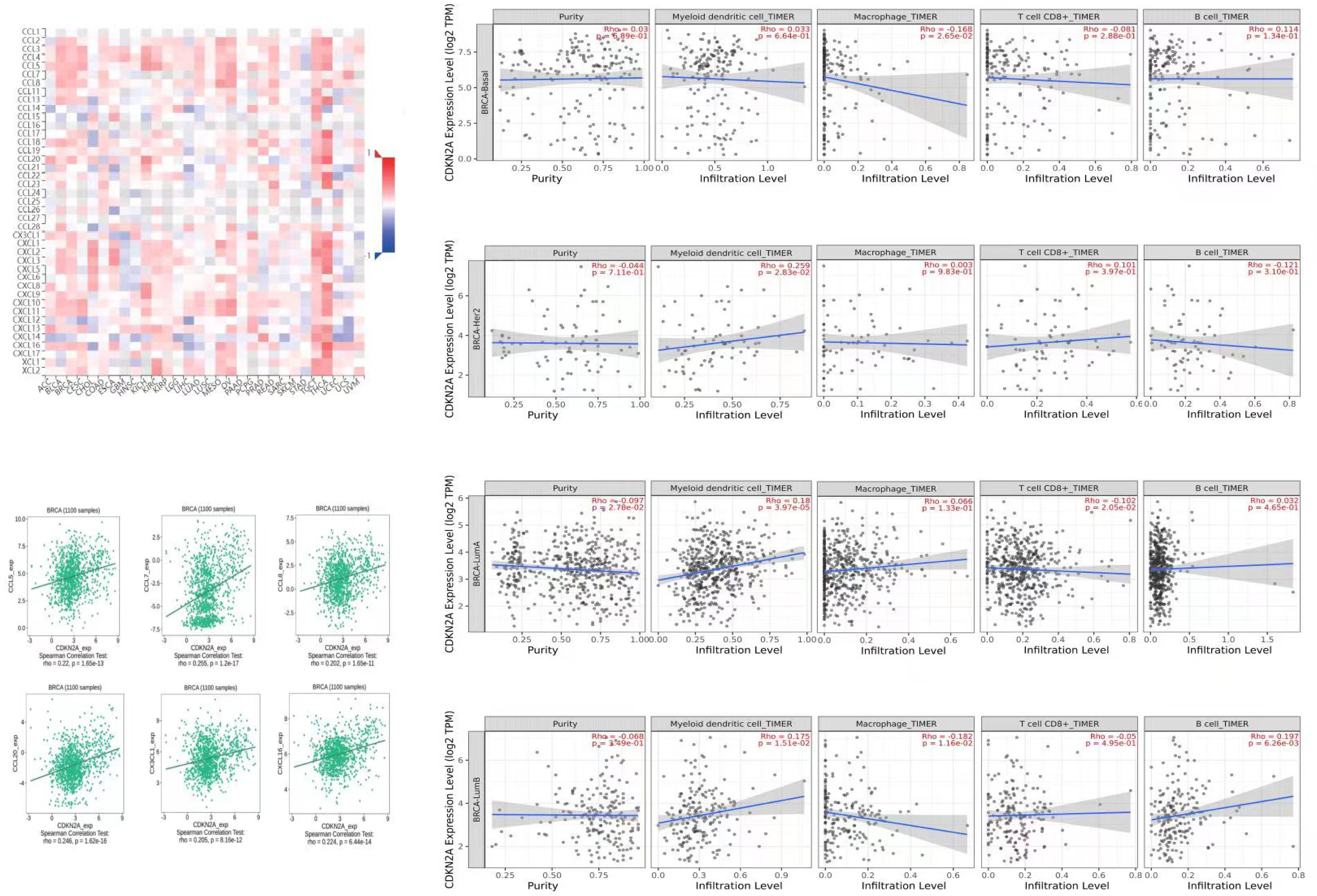

Supplement: Supplementary file 3 [file Image_3.jpeg]

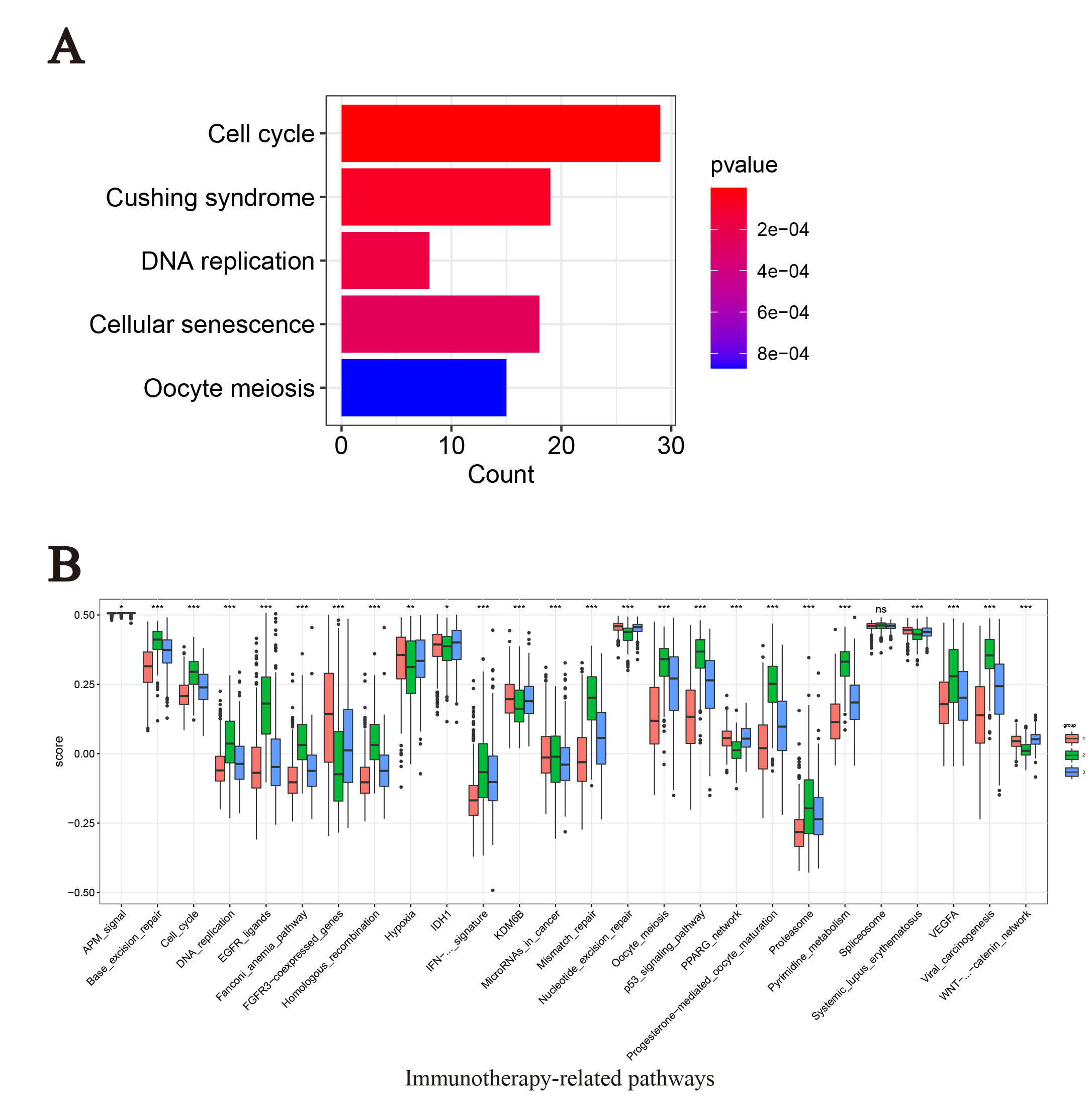

Supplement: Supplementary file 4 [file Image_4.jpeg]

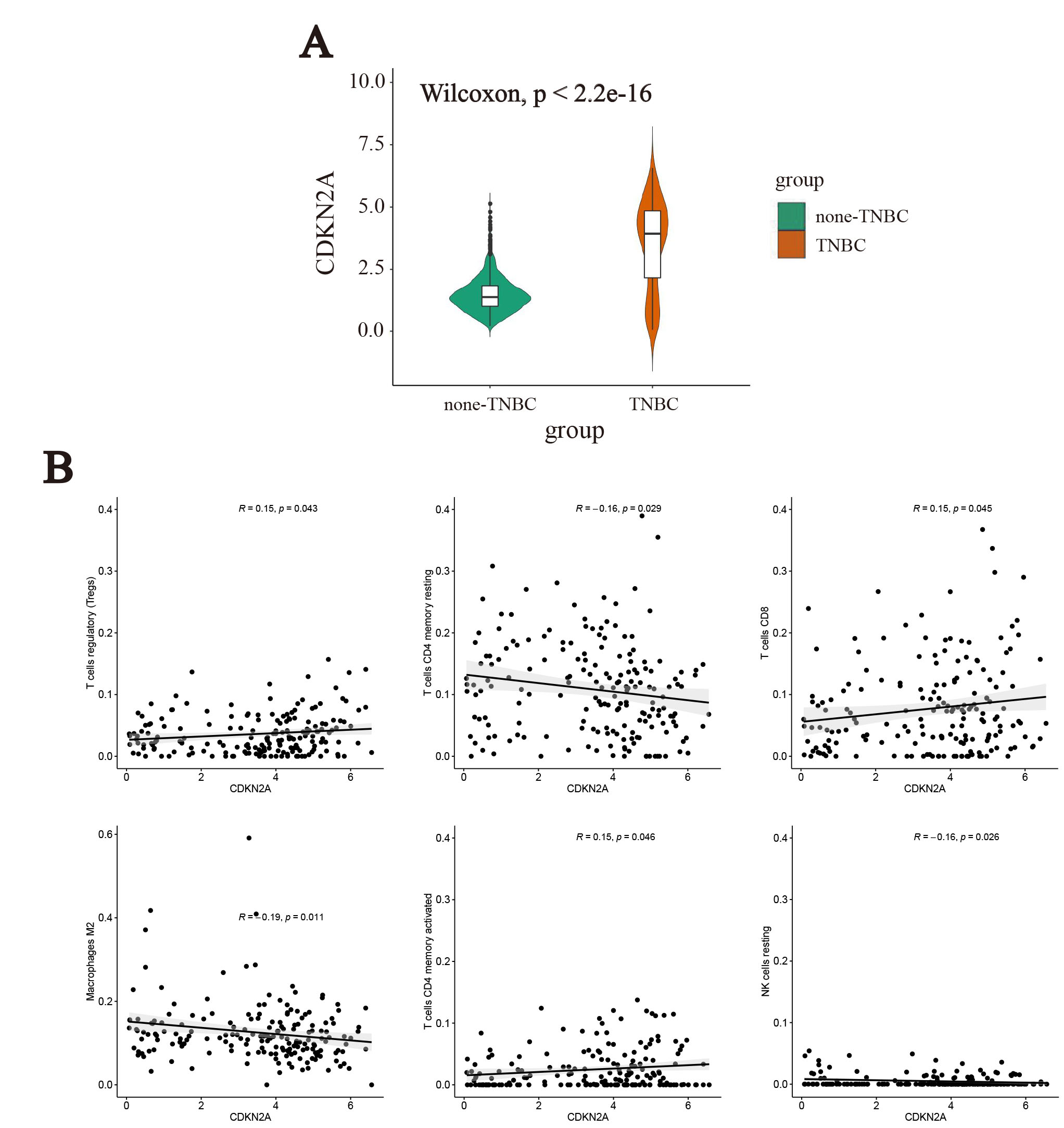

Supplement: Supplementary file 5 [file Image_5.jpeg]

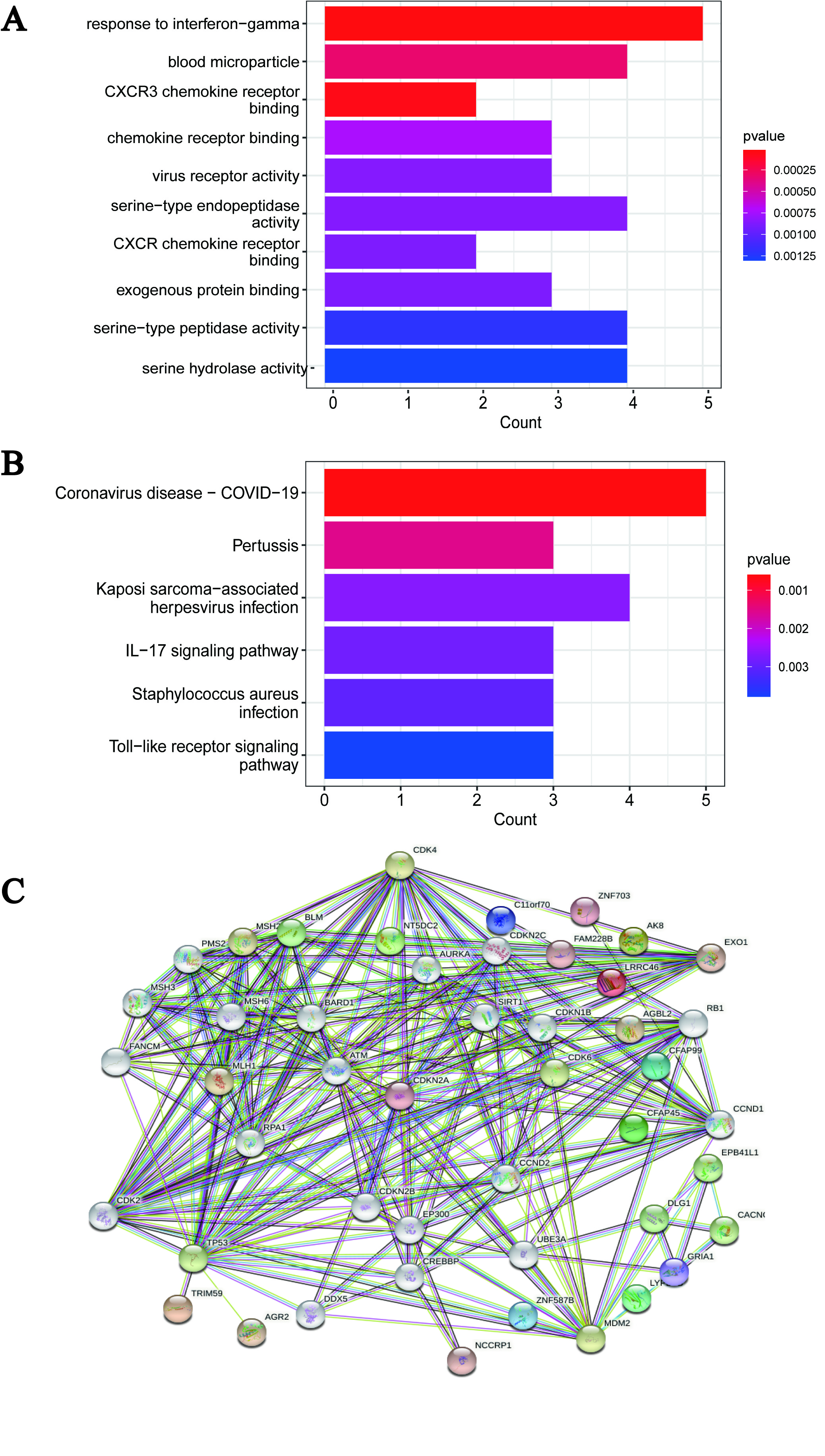

Supplement: Supplementary file 6 [file Image_6.jpeg]
